# In Silico Characterisation of the Late Embryogenesis Abundant (LEA) Protein Families and Their Role in Desiccation Tolerance in *Ramonda serbica* Panc

**DOI:** 10.3390/ijms23073547

**Published:** 2022-03-24

**Authors:** Ana Pantelić, Strahinja Stevanović, Sonja Milić Komić, Nataša Kilibarda, Marija Vidović

**Affiliations:** 1 Laboratory for Plant Molecular Biology, Institute of Molecular Genetics and Genetic Engineering, University of Belgrade, Vojvode Stepe 444a, 11042 Belgrade, Serbia; anapantelic@imgge.bg.ac.rs (A.P.); stevanovic@imgge.bg.ac.rs (S.S.); 2 Department of Life Science, Institute for Multidisciplinary Research, University of Belgrade, Kneza Višeslava 1, 11000 Belgrade, Serbia; sonjamilic@imsi.rs; 3 Department of Pharmacy, Singidunum University, Danijelova 32, 11000 Belgrade, Serbia; nkilibarda@singidunum.ac.rs

**Keywords:** 3D protein structure modelling, de novo transcriptome assembly, differentially expressed gene analysis, drought, intrinsically disordered proteins, liquid–liquid phase separation, resurrection plants, secondary structure prediction

## Abstract

*Ramonda serbica* Panc. is an ancient resurrection plant able to survive a long desiccation period and recover metabolic functions upon watering. The accumulation of protective late embryogenesis abundant proteins (LEAPs) is a desiccation tolerance hallmark. To propose their role in *R. serbica* desiccation tolerance, we structurally characterised LEAPs and evaluated LEA gene expression levels in hydrated and desiccated leaves. By integrating de novo transcriptomics and homologues LEAP domains, 318 *R. serbica* LEAPs were identified and classified according to their conserved motifs and phylogeny. The in silico analysis revealed that hydrophilic LEA4 proteins exhibited an exceptionally high tendency to form amphipathic α-helices. The most abundant, atypical LEA2 group contained more hydrophobic proteins predicted to fold into the defined globular domains. Within the desiccation-upregulated LEA genes, the majority encoded highly disordered DEH1, LEA1, LEA4.2, and LEA4.3 proteins, while the greatest portion of downregulated genes encoded LEA2.3 and LEA2.5 proteins. While dehydrins might chelate metals and bind DNA under water deficit, other intrinsically disordered LEAPs might participate in forming intracellular proteinaceous condensates or adopt amphipathic α-helical conformation, enabling them to stabilise desiccation-sensitive proteins and membranes. This comprehensive LEAPs structural characterisation is essential to understanding their function and regulation during desiccation aiming at crop drought tolerance improvement.

## 1. Introduction

Climate changes will increase the frequency of extended drought periods within the next decades worldwide (https://www.c2es.org/content/drought-and-climate-change/, accessed on 8 February 2022). Drought is a major cause of massive economic losses in agriculture. The success of biotechnological strategies intended to improve crop drought tolerance depends on getting knowledge on the molecular mechanisms required for drought endurance [1].

Among vascular plants, vegetative tissues of a small group of remarkable, collectively called resurrection plants, are recognised as desiccation-tolerant [2]. Resurrection plants can survive in an almost completely dehydrated state (up to 98% of their water content) for months without irreparable damage and can fully re-establish metabolic functions upon rehydration [3]. Since they exhibited the most extreme plant response to water stress (together with seeds), resurrection plants serve as an exceptional research model to improve drought tolerance in crops [1]. *Ramonda serbica* Panc. [4,5] belongs to *Gesneriaceae*, a family that encompassed few other resurrection species (*Haberlea rhodopensis* and *Boea hydrometrica*) extensively described in the literature [6,7,8]. From the evolutionary aspect, as an endemic and tertiary relict [9], *R. serbica* is an excellent model to study vegetative desiccation tolerance, a phenomenon that is considered a critical step in the evolution of primitive land plants [2].

Desiccation or extreme water loss (5–10% of relative water content) leads to protein denaturation, aggregation, and degradation. It affects the fluidity of membrane lipids resulting in loss of membrane integrity at the cellular level [1]. Besides osmotic stress, desiccation provokes the accelerated generation of reactive oxygen species (ROS), e.g., superoxide anion, hydrogen peroxide (H_2_O_2_), and the most toxic hydroxyl radical (HO^•^) [10]. Photosynthesis and respiration are particularly susceptible to oxidative stress during drying [11].

A hallmark of desiccation tolerance is the accumulation of protective late embryogenesis abundant proteins (LEAPs), which may stabilise the correct structure of proteins and membranes during cellular dehydration [3,12,13]. In-depth studies and characterisations of LEA protein families have been carried out in various plants such as Arabidopsis [14], upland cotton [15], potato [16], common wheat [17], tea plant [18], rice [19], pearl millet [20], *Sorghum bicolor* [21], legumes [22], and desert plant *Cleistogenes songorica* [23]. An identification and detailed structural and functional analysis of LEA proteins of resurrection plant species *R. serbica* has not been done yet.

LEA proteins were first discovered 40 years ago in cotton (*Gossypium hirsutum*) seeds, and although they have originally been found to participate in the late stages of seed maturation, they are also expressed in vegetative plant tissues following drought, salinity, and cold stress [12,24,25,26,27]. Moreover, they were described in desiccation-tolerant bacteria and invertebrates (rotifers, nematodes, and brine shrimps) [14,28].

Intrinsically disordered proteins (IDPs) represent a structural class of proteins that do not exhibit well-defined tertiary structures in several regions or throughout the entire sequence [29]. The disorder propensity increases with a higher portion of disorder-promoting amino acid residues (charged: Arg, Lys, Glu, and Asp; structure-breaking: Pro and Gly; and hydrophilic: Ser, Gln, and Asn) in comparison to order-promoting, hydrophobic residues (Trp, Cys, Tyr, Ile, Phe, Val, Ala, and Leu) [30]. Most LEAPs are rich in polar amino acids and predicted to be IDPs [31,32]. These findings are based on various computational algorithms for secondary structure predictions, experimentally verified only in a few cases. The majority of LEAPs are similar in high hydrophilicity and disorder allowing them to adopt a random conformation in aqueous solutions, which turns into an α-helical structure during dehydration [31,32,33,34].

At this moment, no specific physiological function was attributed to LEAPs [3,35]. Their high structural plasticity allows them to interact with various ligands and partners. Studies conducted on several recombinantly produced LEAPs from different species, including *Arabidopsis thaliana*, have suggested that LEAPs can be involved in water binding, ion sequestration, stabilisation of membranes and enzymes during freezing or drying [33,36,37,38,39]. Due to their structural plasticity, it is considered that LEAPs can act as “molecular shields” and affect protein aggregation [40,41,42]. Accordingly, two hypotheses were proposed. Firstly, as shield molecules, LEAPs can physically separate cellular entities from each other during desiccation to omit crowding-promoting formation of protein aggregates [43]. Secondly, due to structural plasticity, LEAPs can directly interact with their specific target proteins, making them more stable during water reduction [13,31,40,44].

On the other hand, IDP-induced liquid–liquid phase separation (LLPS) is a mechanism by which non-membranous organelles, i.e., intracellular proteinaceous condensates, are created [1,29]. Recently, it was suggested that LEAPs increase cells’ structural integrity and intracellular viscosity during desiccation by forming separate intracellular proteinaceous condensates [28,45]. However, the details regarding the recruitment of specific or nonspecific target/client proteins and the importance of physical separation within the proteinaceous condensates among various cellular components are still debatable [3]. The influence of the microenvironment (i.e., pH, osmotic potential, and ionic strength); presence of other solutes (sucrose and raffinose); and posttranslational modifications on specific LEAP disorder-to-order transitions and, therefore, functions are still elusive [35].

Taken together, the molecular mechanism of a broad range of proposed stabilisation strategies remains unclear, and the mechanism underlying the protective effects of LEAPs on cellular components under dehydration (freezing, desiccation, and osmotic stress) is still unexplained. Structural characterisation of LEAPs is a key to understanding their function and regulation of their intrinsic structural disorder-to-order transition during desiccation.

The aim of our study was to identify, characterise, and estimate potential role of *R. serbica* LEAPs in desiccation tolerance. To achieve these objectives, we performed a de novo transcriptome analysis of *R. serbica* and analysed differentially expressed genes encoding LEAPs in hydrated (HL) and desiccated leaves (DL). We emphasised the similarities within and differences between seven LEA protein family groups in physicochemical properties, amino acid composition, conserved structural motifs, secondary structure, subcellular localisation, and correlated the observations with the expression level of LEA genes in HL and DL. The obtained results will pave the way for identifying LEAPs endogenous partners and their target molecules in the cell, giving more insights into protective mechanisms of desiccation tolerance aiming at improving crop drought tolerance.

## 2. Results

### 2.1. Identification and Classification of R. serbica LEAPs

Previously, we performed transcriptomic analysis of *R. serbica* hydrated leaves (HL) gene expression under regular watering conditions [46]. Since our aim was to identify and characterise desiccation-induced late embryogenesis abundant (LEA) genes in *R. serbica* leaves, we improved our database and expanded it on desiccated leaves (DL). The completed *R. serbica* de novo transcriptome database is available at: https://zenodo.org/record/6341873#.YijgJ_7MJPY, accessed on 22 March 2022 (10.5281/zenodo.6341873) and translated into amino acid sequences at: https://zenodo.org/record/6340979#.YiitWP7MJPY, accessed on 22 March 2022 (10.5281/zenodo.6340979). The sequence data from this article can be found in the Short Read Archive database at NCBI under accession numbers SRR18015613 and SRR18015612 (bioproject accession no. PRJNA806723 and sample accession no. SAMN25859880). Overview of the data production quality, length distribution, and number of transcripts and unigenes and annotated unigenes is given in Supplementary Materials Table S1. In total, 49.1% of annotated sequences showed the best matches with *B. hygrometrica* Bunge. R. Br. (homotypic synonym: *Dorcoceras hygrometricum*) sequences (Supplementary Figure S1).

The NCBI NR protein database search of the obtained merged transcripts of both HL and DL using Basic Local Alignment Search Tool (BLAST) listed 433 members of the LEA gene family (Supplementary Table S2). The obtained *R. serbica* LEAPs sequences were highly homologous with *Striga asiatica* LEAPs, followed by *Capsicum annuum* (Supplementary Figure S2). Almost 20 hits were related to LEAPs identified in *D. hygrometricum*. The final set of 318 *R. serbica* LEAPs was created upon removing proteins consisting of less than 100 amino acids from the list of 359 LEAPs containing LEA domains (Supplementary Table S2).

According to the annotated LEA domains, all identified LEAPs were grouped into seven protein family groups, ranging from LEA1 to LEA5, dehydrins, and seed maturation proteins (SMPs), as adopted by Reference [14] (Supplementary Table S2). The most populated *R. serbica* LEA protein family group was LEA2, containing 127 proteins (almost 40% of the total identified LEAPs), followed by LEA4, which encompassed 96 proteins (~30%), while the smallest group, LEA5, included 11 proteins (Table 1).

The phylogenetic analysis revealed that proteins belonging to the same LEA protein family group were phylogenetically related, with at least one clade with a common node. The exception was twenty-five LEA4 protein family group members that belonged to separate, independent clades (Supplementary Figure S3). These proteins were evolutionary the most distant from the LEA2 proteins, as indicated by their positions on the opposite sides of the unrooted tree. In total, a hundred closely related gene pairs/paralogues were observed within all LEA groups.

To determine the homology of *R. serbica* LEA protein family groups with those well-annotated in *A. thaliana* [14] and in upland cotton [15], multiple sequence alignment (MSA) within the respective LEA protein family groups was done (Table 1). Phylogenetic analyses indicated the highest sequence homology between *R. serbica* and *A. thaliana* LEAPs within the LEA5 protein family group (~60%) (Table 1). Regarding the sequence similarities between *G. hirsutum* and *R. serbica* LEAPs, the highest value, almost 34% homology, was detected for dehydrins.

### 2.2. Physicochemical Analysis of R. serbica LEAPs

The physicochemical characteristics (like sequence length, pI, amino acid composition, protein’s molecular weight, and grand average hydropathy—GRAVY) for all *R. serbica* LEAPs are tabulated in Supplementary Table S2. The *R. serbica* LEA proteins were observed to have variable amino acid sequence lengths up to 444 aa (LEA4 protein group) corresponding to molecular weight of 44.9 kDa. The average sequence length of the *R. serbica* LEA2 family members was the highest (~226 aa), followed by LEA4 (~187 aa), while LEA5 proteins were the shortest (118 aa) (Table 2). Members of the LEA4 protein family group were observed to exhibit the most variable sequence lengths and molecular weights (Supplementary Materials Table S2).

**Figure 1:**
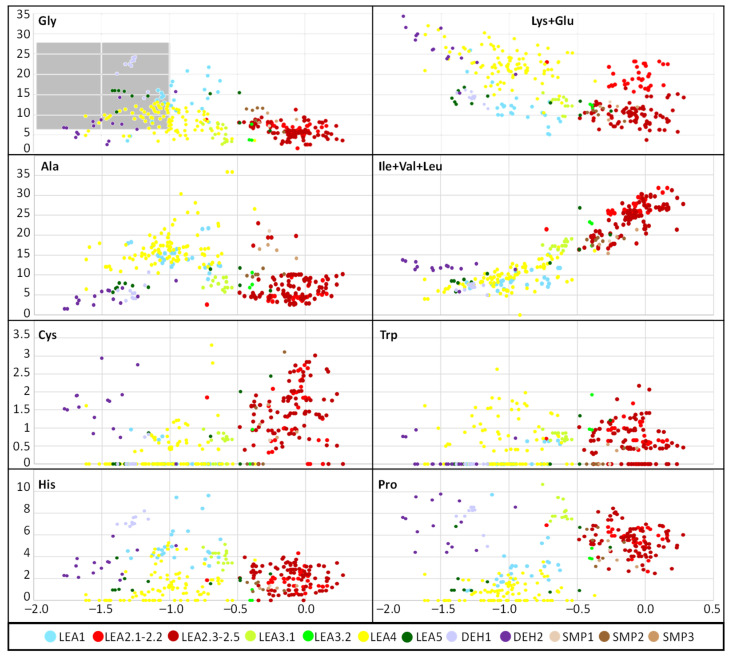
Percentage of selected amino acids and Gly versus GRAVY index plot in *R. serbica* LEA protein family members. The distribution of hydrophilins is highlighted in grey in the Gly/GRAVY plot.

The LEA2 and LEA1 protein family group members were the most basic, with the average pI = 8.2–8.4, while the SMPs were mostly acidic, with an average pI value of 4.9 (Table 2). The GRAVY index values were negative for all *R. serbica* LEA proteins, except for some members of the LEA2 protein family group, although the average GRAVY value for the most hydrophobic group was −0.09 (Table 2). The calculated GRAVY indices indicated that *R. serbica* dehydrins were the most hydrophilic, showing the most negative GRAVY index, followed by LEA5 and LEA4 (Figure 1).

The average amino acid composition of each *R. serbica* LEA protein family group is presented in Table 2 and Supplementary Table S2. The percentage of cysteine was generally low in the identified LEAPs. It was the highest within the LEA2 protein group members and one half of dehydrins and lowest in proteins belonging to the LEA1 and LEA4 groups (Figure 1 and Supplementary Table S2). The charged amino acid content was the highest in dehydrins, followed by LEA4 proteins (Table 2). Accordingly, dehydrins and LEA4 family members contained the highest content of Lys (13–17%), Glu (up to 18%), and Asp (up to 10%) compared with the other LEA protein groups. The contents of aliphatic residues (Ile, Val, and Leu) were the highest in the LEA2 protein family group members, followed by SMPs in the case of Val. The exceptionally high content of alanine residues (up to 20%) was found in the LEA4, SMP, and LEA1 protein group members. Proline was the most abundant in dehydrins and LEA3 proteins. The histidine percentage was the greatest in dehydrins, followed by LEA1 protein family members (Figure 1). Among *R. serbica* LEAPs, the glycine content was the highest in DEH1, LEA1, and LEA5 protein family groups. The tryptophan content was generally low among *R. serbica* LEAPs; it was the greatest in proteins belonging to the LEA3, LEA2 and LEA4 protein families (~1% of the total sequence length).

### 2.3. Homology Motifs Analyses of R. serbica LEAPs

**Figure 2:**
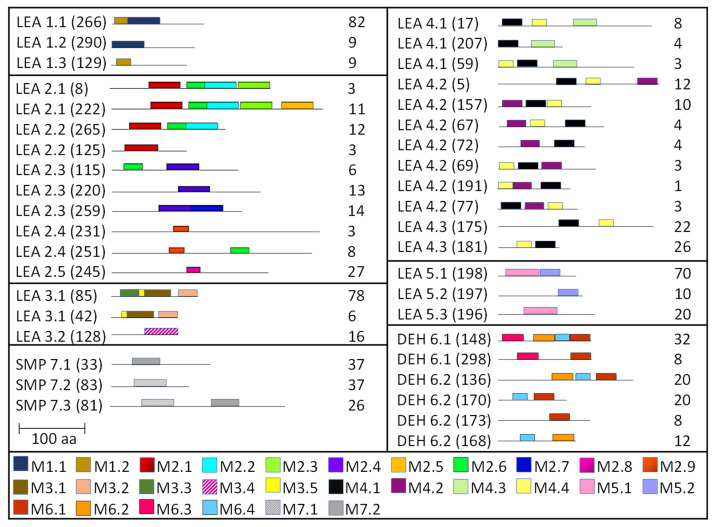
MEME motifs and motif logos of representative LEAPs of each *R. serbica* LEA protein family group and subgroup. The numbers in the parentheses present the RsLEAP code (see Supplementary Tables S2 and S3). The consensus and logo sequences of each motif are presented in Table 3. The numbers at the end of each protein sequence present a percentage of LEAPs with the same motif pattern in the respective LEA protein family group. The bar represents 100 aa.

To gain more information regarding structural diversity and conserved motif divergence of LEAPs from *R. serbica* within seven distinctive LEA protein family groups, a domain architecture analysis was performed. Supplementary Figures S4–S10 present conserved motif composition analysis of each LEAP (318) from *R. serbica* within the specific LEA protein family group. To simplify the presentation and stress particular differences among the groups, the representatives with unique motif patterns were selected and are presented in Figure 2.

The LEA1 protein family group includes LEAPs similar in length (Supplementary Table S2) that mostly contained (80%) two highly conserved motifs: M1.1 and M1.2 (Supplementary Figure S3) (Figure 2). Both motifs were recognised as “LEA1” protein family domains by the Pfam database. Members of this protein family group could be clustered into three subgroups: LEA1.1 (M1.1 and M1.2 motifs), LEA1.2 (M1.1 motif), and LEA1.3 subgroup (M1.2 motif). The first, M1.1 motif contained 50 aa, with three conserved Lys residues present in almost all LEA1 members and seven highly conserved alternating Glu/Asp residues (Table 3). In addition, the M1.1 consensus sequence contained 20 charged of the 50 total residues, with a GRAVY index that indicated the M1.1 motif was very hydrophilic (Table 3). The second M1.2 motif encompassed 21 aa, including two conserved Lys, two Glu, one Gly, and seven Ala residues.

According to the homologous motifs, the LEA2 family was clustered into five major subgroups: LEA2.1, LEA2.2, LEA2.3, LEA2.4, and LEA2.5. Nine motifs were identified in this LEA group (Supplementary Materials Figure S5 and Table 3). Motifs M2.2, M2.3, M2.4, M2.5, and M2.8 contained “LEA2” protein family domains, according to the Pfam database. Subgroup LEA2.2 contained motifs M 2.1, M2.2, and M2.6, while the “extended” subgroup LEA2.1 contained two additional motifs: M2.3 and M2.5. Additionally, all LEAPs belonging to subgroup LEA2.3 encompassed the M2.4 motif, while members of two clusters within this subgroup contained additional motifs M2.6 or M2.7 (Figure 2). Motifs M2.6 and M2.7 with dominant nonpolar residues were the most hydrophobic among all the motifs detected in *R. serbica* LEAPs (Table 3). Motif M2.9 was a determinant motif for the subgroup LEA2.4, although some members of this subgroup contained motif M2.6 as well (Figure 2). Proteins within the subgroup LEA2.5 differentiated from the other LEA2 protein members by the presence of the M2.8 motif (Supplementary Figure S5).

The LEA3 protein family group was clustered into two subgroups: LEA3.1 and LEA3.2 (Figure 2). With the exception of RsLEA_42, four highly conserved motifs (M3.1, M3.2, M3.3, and M3.5) were found in the LEA3.1 group (Supplementary Materials Figure S6). Interestingly, motifs M3.1 and M3.2 were rich in proline and glycine residues and contained almost ten completely preserved charged amino acids (Table 3). In addition, motif M3.1 contained a conserved Trp residue. Motif M3.3 (29 aa) was rich in Ser, Arg, and aliphatic amino acids, similar to motif M3.2, while, in short, motif M3.5 (6 aa) Arg, His, and Val were the dominant residues. Three LEA3.2 proteins contained a single motif M3.4 recognised as the “LEA3” protein domain, according to the Pfam database (Table 3).

Four distinctive motifs were identified in the LEA4 protein family group (Supplementary Figure S7 and Table 3). All members of the LEA4 protein family group contained the M4.1 motif, rich in charged amino acid residues. Subgroup LEA4.1 members contained the most polar *R. serbica* LEA motif M4.3 (GRAVY index = −1.96), LEA4.2 contained motif M4.2, and all other LEA4 protein members were nested into LEA4.3 (Figure 2 and Supplementary Figure S7). Except M4.2, all motifs identified in the LEA4 protein family group were very polar and rich in charged amino acid residues (>50%), namely lysine (20–33 %) (Table 3). Indeed, almost a quarter of the *R. serbica* LEA4 protein group members contained at least one of the motifs from the KYS and Lys-rich motif classification system (Supplementary Table S4). Based on the Pfam database, “LEA” protein domains were found in motifs M4.1 and M4.4.

Only 11 LEAPs formed the *R. serbica* LEA5 protein family group (Supplementary Table S3 and Supplementary Figure S8), among which nine LEAP encompassed the highly conserved motif M5.1 (Figure 2 and Table 3). In eight LEAPs of this group, motif M5.2 was found with strongly preserved glycine and charged residues (Table 3). Pertinent to that, the GRAVY index of these two motifs (almost −1.2, Table 3) indicated their high polarity.

Based on the motif homology, dehydrins were clustered in two subgroups: DEH1 and DEH2, which contained four distinct polar motifs (M6.1–M6.4) (Figure 2). Members of the DEH1 subgroup were determined by the motif M6.3, rich in glycine, proline, and tyrosine residues and negatively charged amino acids (Figure 2 and Table 3). This motif contained the commonly called Y-segment DEYGNP (Table 3). Almost 84% of the *R. serbica* dehydrins contained motif M6.1, encompassing the commonly called K-segment: KKG[_N][MF]M[DE]KIKEK (Table 3 and Table S4). The greatly conserved motif M6.2 was prevalently composed of eight Ser (so-called S-segment), six Gly, three Pro, and eight charged residues (Table 3). In *R. serbica* dehydrins, the prevalent S-segment was SGSSSSSSS (namely in the DEH1 protein subgroup), although the S7, S8, and TGSSSSSS motifs were detected as well (Supplementary Materials Table S4). Conserved motif M6.4 contained mainly charged amino acids, Pro, and Gly, similar to other motifs in this family group. Motifs M6.1, M6.2, and M6.4 encompassed the “dehydrin” protein family domain, as indicated by the Pfam database. Taken together, all dehydrins identified in *R. serbica* contained at least one dehydrin-determining segment (Supplementary Table S4).

Seed maturation proteins (SMPs) were clustered into three subgroups: SMP1, SMP2, and SMP3, depending on the presence and absence of two detected motifs: M7.1 and M7.2 (Figure 2 and Supplementary Materials Figure S10). Motif M7.1 involved seven fully preserved alanine, three valine, and four glycine residues, as well as five negatively charged residues, in all proteins. The shorter motif M7.2 encompassed mostly aliphatic (namely, Ala and Val) residues, leading to an almost positive GRAVY index. Both motifs were recognised as “SMP” protein family domains by the Pfam database.

### 2.4. Structure and Disorder Prediction of R. serbica LEAPs

*R. serbica* LEAPs significantly differ in their secondary structure, disorder propensity, and aggregation potential between distinct LEA protein family groups (Supplementary Table S3). Five secondary structure predictors showed that more than 30% of the identified *R. serbica* LEAPs exhibited a high propensity to form α-helices (>70% of the total sequence length), while almost 35% of all identified *R. serbica* LEAPs showed the potential to form β-sheets in at least 30% of their sequence length. Almost 25% of LEAPs found in *R. serbica* leaves exhibited a propensity to organise at least 50% of their sequence in the form of a random coil.

**Figure 3:**
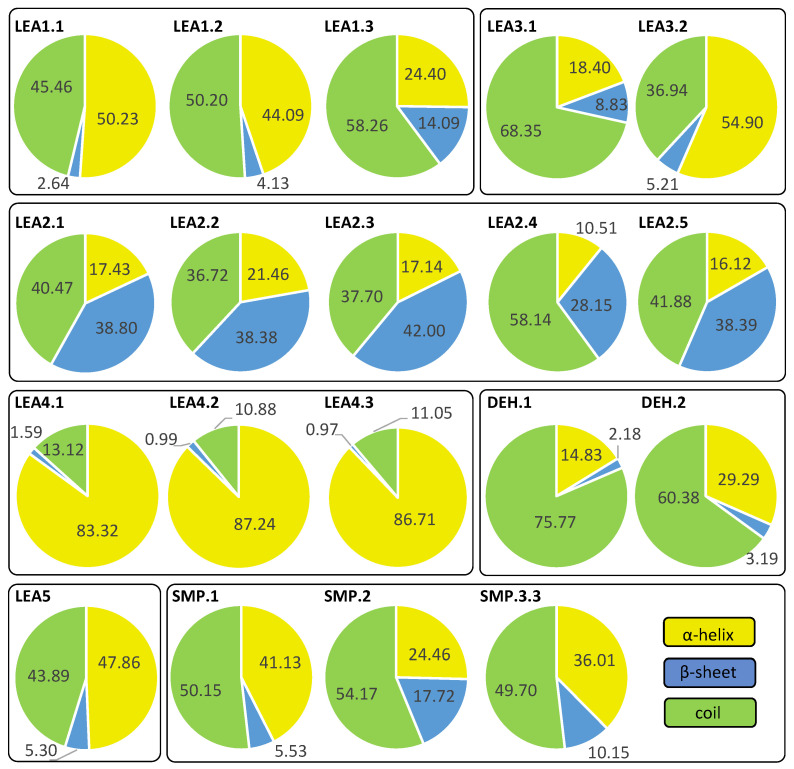
Average distribution of the predicted secondary structure of each *R. serbica* LEA protein family group and subgroup members according to the five secondary structure prediction algorithms: PsiPred, Sopma, FELLS, Phyre2, and JPred4.

Particularly, the LEA4 protein family group exhibited a high propensity to form α-helices (in the range 71–97% of the sequence length). On average, only ~1% of the *R. serbica* LEA4 family members sequence was predicted to form β-sheets (Figure 3). In addition, a very low propensity for adopting β-sheet conformation (up to 5% of the sequence length) exhibited also members of dehydrins, and the majority of LEA1 protein family. On the contrary, the LEA2 family group, particularly the LEA2.3 subgroup, showed a high potential to form β-sheets and a low propensity for α-helices (Supplementary Table S3). The positive correlation between the percentage of the sequence predicted to adopt a random coil and the sequence length among the LEA2 protein family subgroups was noticed. For example, members of the LEA2.4 subgroup with an average length of 298 aa exhibited a propensity to undergo random coil conformation for 58% of the sequence length. The prevalent conformation observed in the members of dehydrins (particularly, the DEH1 subgroup, 76% of total sequence length), LEA3 (63%), and SMP (51%) family groups was random coil (Supplementary Table S3).

**Figure 4:**
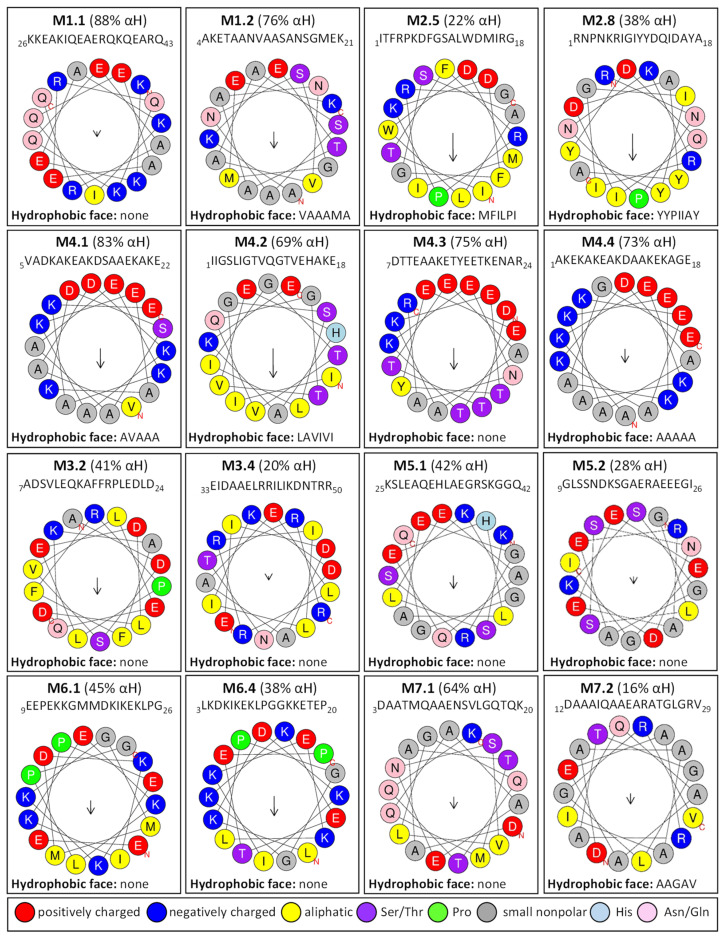
Modelling of the α-helix structure within detected MEME motifs in R. serbica LEA protein family members. Helical projections of α-helices were generated using the HeliQuest webserver [47]. αH; predicted α-helix percentages obtained by FELLS. Each wheel was obtained with an 11-amino acid window. The arrow shows the helical hydrophobic moment.

To get more information regarding α-helices within *R. serbica* LEAPs, the structural properties of the obtained protein motifs (Table 3) were analysed. Motif M1.1 intended to form a charged α-helix, with distinctive positive and negative faces, while, in M1.2, a hydrophobic face was also proposed (Figure 4). Motifs M2.5 and M2.8 exhibited a low tendency to adopt amphipathic α-helical structures. In the *R. serbica* LEA3 protein family group, the only motifs predicted to form an α-helical structure were M3.2 and M3.4, but no hydrophobic face was modelled. All four motifs in the LEA4 protein family group were predicted to be organised as α-helices (Figure 4). According to the HeliQuest results, they all, except the M4.2 motif, contained negatively charged faces, while motifs M4.1, M4.2, and M4.4 exhibited hydrophobic faces as well (so-called A type of the α-helix). On the contrary, motifs M5.1 and M5.2 showed a lower tendency to form α-helices with no hydrophobic faces (Figure 4). Only two motifs characteristic for *R. serbica* dehydrins, M6.1 and M6.4, were predicted to form α-helices, while, in the motifs M6.2 and M6.3, the dominant conformation for more than 94% of the total sequence length was the random coil. In addition, both motifs identified in the SMP family group were predicted to form α-helices. Moreover, M7.2 tended to form a hydrophobic face (Figure 4).

Surprisingly, despite a low propensity for folding into α-helical conformation, the presence of at least one transmembrane α-helix (TMH) within the *R. serbica* LEA2 protein family was predicted both by TMHMM and FELLS predictors (Supplementary Table S3). For example, almost all LEAPs belonging to the subgroups LEA2.3–5 were predicted to form at least one TMH comprised of approximately 20 amino acids, while, in only two protein members of both the LEA2.1 and LEA2.2 groups, a single distinctive TMH was observed. In addition, in seven LEA2.3 group protein members, the additional TMH (two in total) was observed. In total, 32 different and hydrophobic TMH domains were identified in 87 TMH-containing proteins belonging to the LEA2 protein family group (Supplementary Table S5 and Supplementary Figure S11). On the other hand, members of the SMP, dehydrin, LEA1, LEA3, and LEA5 protein family groups were predicted to be soluble—no transmembrane domains were predicted (Supplementary Table S3).

Besides these three elements of protein secondary conformation, we analysed the disorder propensity of the identified *R. serbica* LEAPs. As predicted by several bioinformatic tools, more than 55% of the identified LEAPs were found to be disordered (>50% of the sequence length) (Supplementary Table S3). Indeed, more than 92% of the *R. serbica* LEAPs (with the exception of the LEA2 protein group) exhibited a propensity to be disordered.

Comparisons between seven *R. serbica* LEA protein family groups showed that, on average, dehydrins (particularly, DEH1 members) and LEA1 exhibited the highest propensity for the disorder (87–97% of the total sequence length), followed by LEA4 (80–83%) of the total sequence length) and LEA5 (79%) (Supplementary Table S3). On the contrary, members of the LEA2 protein family group showed the highest hydrophobic effect and the lowest disorder propensity (22% of the sequence length), except in the case of the LEA2.4 subgroup, where the disorder propensity was twice higher.

These findings were positively correlated with the predicted number and size of the globular domains (Supplementary Table S3). All the *R. serbica* LEA2 family members were predicted to form a single globular domain, occupying between 94 and 96% of the sequence length in the case of all LEA2 groups, except the LEA2.4 protein subgroup. On the contrary, no globular domain was predicted among all dehydrin, LEA1, and LEA4.1 protein members. Within the LEA4.2 protein subgroup, 11 of the 35, and within the LEA4.3 subgroup, 7 of the 47 members were predicted to fold into a single globular domain. Almost 83% of the LEA3 protein family members were predicted to fold into a single globular domain, while 35% of *R. serbica* SMPs were predicted to be organised into one or two globular domains.

**Figure 5:**
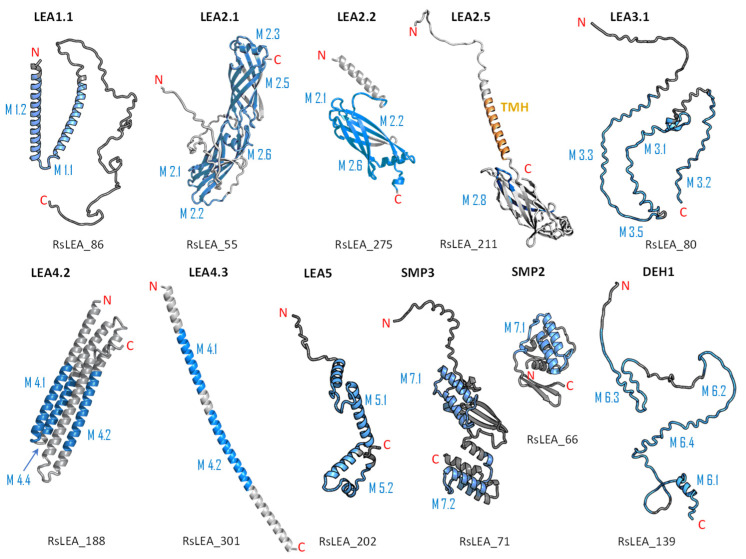
Three-dimensional models of the representative LEAPs of each *R. serbica* LEA protein family group. Detected MEME motifs are denoted in blue. The RsLEA code for each protein is given. Orange: transmembrane α-helix, TMH.

The obtained information derived from the representative structural model is the key to understanding the function of LEAPs and the regulation of their intrinsic structural disorder-to-order transition during desiccation. Therefore, to incorporate all the structural findings and predictions, we constructed 3D models with prediction quality of the representatives of seven LEA protein members (Figure 5 and Supplementary Figure S12).

As already presented, in the RsLEA_86 protein, a member of the LEA1 protein group, two distinctive α-helices encompassing the M1.2 and M1.1 motifs at the N-terminus and a random coil at C-terminus were obtained. Sixteen members of the LEA2.1 were presented with the RsLEA_55 protein, containing M2.1, M2.2, M2.3, M2.5, and M2.6 organised in two successive β-barrel domains at the C-terminus and N-terminal random coil (Figure 5). For all members of the LEA2.3, LEA2.4, and LEA2.5 protein family subgroups, a hydrophobic TMH followed by a globular β-barrel structural domain was shown on the example of RsLEA_211 (Figure 5). The difference in the structures of the proteins belonging to the mentioned subgroups was related to the N-terminal random coil, whose length varied in relation to the whole protein sequence length. In addition, the LEA2.2 protein subgroup members, represented by RsLEA_275, also folded into a β-barrel structural domain at the C-terminus and N-terminal α-helix, composed of 20 residues, similar to the shorter members of the LEA2.3–2.5 subgroups (Figure 5). In contrast to these proteins, in the RsLEA_275 protein, this α-helix was amphipathic, composed of a hydrophobic face and more polar residues, resulting in a net charge of +3, due to the presence of four lysin, one arginine, one glutamate, and one aspartate residue.

Besides the LEA1 and LEA2 protein family groups, a good correlation between the presented results and constructed 3D models was also obtained for the dehydrins, SMPs, LEA4, and LEA5 groups (Figure 5). High disorder and random coil propensities were characteristic for dehydrins, evidenced by the 3D model of the representative RsLEA_139, and obtained higher predicted alignment error (PAE) values (Supplementary Figure S12). Structural differences within the *R. serbica* SMPs were illustrated by two representatives, a shorter RsLEA_66 containing only the M7.1 motif, compactly folded into one globular domain composed of all three secondary structure elements and a longer RsLEA_71 containing both the M7.1 and M7.2 motifs, and the N-terminal random coil. The exceptionally high propensity for folding into an α-helical conformation, particularly an A-type α-helix (HeliQuest, data not shown), was demonstrated for the *R. serbica* LEA4 protein members, e.g., RsLEA_188 and RsLEA_301 (Figure 5).

An almost equal distribution of α-helices and coils, with a very low percentage of β-sheets and the absence of a globular domain, was confirmed for the LEA5 family members represented by the RsLEA_202 protein. As a representative of the *R. serbica* LEA3 protein family group, RsLEA_80 mostly folded into a random coil and showed a high PAE value, implicating a significant disorder propensity (Figure 5).

### 2.5. Calculated Hydroxyl Radical Scavenging Ability (HRSA) of R. serbica LEAPs

In our previous work [48], we displayed the antioxidative ability of free proteogenic amino acids through determining their hydroxyl radical (HO^•^, generated in the Fenton reaction: Fe^2+^ + H_2_O_2_ → Fe^3+^ + OH^−^ + HO^•^) scavenging rate by using electron paramagnetic resonance. The obtained hydroxyl radical scavenging abilities (HRSA) were higher for the hydrophobic amino acid residues. The rank order according to the amino acid HRSA was: Trp > Phe, Leu > Ile > His > Arg > Val > Lys, Tyr, Pro > Gln, Thr, Ser > Glu, Ala, Gly, Asn, and Asp. The obtained HRSA for single amino acids were used to calculate the protein HRSA for 318 annotated *R. serbica* LEAPs based on their sequence (Supplementary Materials Table S3).

The obtained HRSA values ranged from 3.9 to 113.7. The highest HRSA was shown for the *R. serbica* LEA2.1 subgroup members (the average HRSA was 95.6), followed by LEA2.4, LEA2.5, and LEA2.3, while the lowest HRSA was accompanied with the members of the LEA1 protein family subgroup (the average HRSA was 16.5), followed by the members of the DEH1, SMP1, LEA5, and LEA4.3 protein family (sub)groups (Supplementary Table S3).

### 2.6. Cellular Compartmentalisation of R. serbica LEAPs

**Figure 6:**
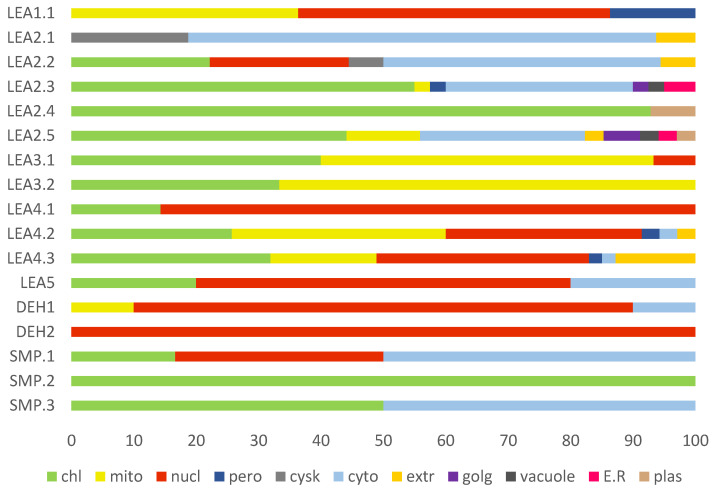
Repartition of the *R. serbica* LEAP-predicted subcellular distribution in each family (sub)group. Results are given in percentages. Chl, chloroplast; mito, mitochondrion; nucl, nucleus; pero, peroxisomes; cysk, cytoskeleton; cyto, cytosol; ext, extracellular space; golg, Golgi apparatus, E.R. endoplasmic reticulum; plas, plastids.

Determination of the subcellular location of a protein is essential to understanding its biochemical function. The majority of LEAPs were predicted to participate in the secretory pathway (Supplementary Table S2). To predict the specific compartmentalisation of each LEAP, the WoLF-PSORT tool was used (Figure 6). Most of the LEAPs from *R. serbica* were predicted to be chloroplastic (98), nuclear (87), cytosolic (52), and mitochondrial (48).

The LEA protein family groups differed also regarding their subcellular compartmentalisation (Figure 6). For example, more than one-third of the members of the LEA1 and LEA3 protein family groups are predicted to be mitochondrial proteins. The majority of LEAPs associated with the LEA1, LEA4.1, LEA5, and dehydrins exhibited a high propensity to be located within the nucleus. Proteins belonging to the SMP2, SMP3, LEA3, LEA2.3, LEA2.4, and LEA2.5 protein family (sub)groups should be found in the chloroplasts. In contrast, more than 30% of the members of the LEA2.1, LEA2.2, LEA2.3, SMP1, and SMP3 protein family subgroups are predicted to be cytosolic proteins (Figure 6). Significantly, eleven annotated *R. serbica* LEAPs are predicted to be localised in the extracellular compartment, and these proteins belonged to the LEA2.1, LEA2.2, LEA2.5, LEA4.2, and LEA4.3 protein family subgroups.

### 2.7. Analysis of Differentially Expressed R. serbica LEA Genes

In total, 88 different genes encoding LEAPs were differentially expressed upon desiccation in *R. serbica* leaves (FDR of <0.05 and log2 (DH/HL) > 2) (Table 4). Among them, 76% were upregulated and 24% were downregulated in DL compared to HL. Within the upregulated LEAP-encoding genes, almost 21% of the encoded proteins belonged to the LEA4.3 protein family subgroup, and almost 14% belonged to the LEA1 protein family group. At the same time, 67%, 63%, and 60% of the members of the protein family subgroups SMP2, LEA5, and DEH1 were upregulated upon desiccation. All differentially expressed genes (DEGs) belonging to the LEA1, LEA2.1, LEA5, dehydrins, and SMP gene family groups were upregulated in desiccated leaves compared with the hydrated ones. On the other hand, most of the downregulated genes encoded proteins associated with the LEA2.3 and LEA2.5 protein family subgroups (15% and 12%, respectively). Within the LEA4.1 gene family subgroups, no DEG was observed (Table 4).

Considering the size of each LEA protein family group and the number of upregulated LEA genes, the LEA5 gene family had the greatest portion within all the increased DEGs upon desiccation, followed by the LEA1, SMP, dehydrins, LEA4, and LEA2 gene family groups.

## 3. Discussion

With the increasing number of plant genomes available, a comprehensive analysis of the evolution and functional diversification of late embryogenesis abundant (LEA) gene families became possible. *Ramonda serbica* is a hexaploid species, with a 1261-Mbp 1C genome size [49], but its genome is not sequenced. Therefore, the prerequisite for identifying LEAPs of hydrated (HL) and desiccated leaves (DL) of *R. serbica* was to obtain an improved and reliable RNA database.

### 3.1. Identification and Classification of R. serbica LEAPs

Recently, we provided the first *R. serbica* transcriptome database, encompassing 47,000 annotated genes, respectively [46]. The presented transcriptome database is significantly improved here, containing approximately four times more newly annotated unigenes and encompassing data related to DL as well.

A significantly higher number of annotated genes was found in *R. serbica* leaves compared with three resurrection plants: *D. hygrometricum* and *H. rhodopensis*, sharing the same family as *R. serbica* [6,7], and *C. plantagineum* [50]. Surprisingly, the homology sequence analysis of the initial 433 annotated LEAPs showed that most of the hits (around 100) belonged to *Striga asiatica* LEAPs, although it is not in close taxonomic positions with *R. serbica* (compared with *D. hygrometricum*). However, *S. asiatica* is a drought-tolerant species that favours relatively dry and infertile soils of semi-arid tropics of Africa and Asia. It is an ABA-insensitive plant that keeps the stomata open even under drought conditions [51]. Unexpectedly, only twenty hits were related to LEAPs associated with resurrection plant *D. hygrometricum* [7]. The reason for that might originate from a poor functional annotation of *D. hygrometricum* genome data, containing a large number of so-called “hypothetical proteins”.

During the last three decades, different authors have separated LEAPs into different groups using different classification criteria [52,53,54]. For a better outline and protein comparison between different species, the most widely employed Pfam nomenclature was used in this study.

The final *R. serbica* LEAP list involved 318 LEAPs organised into seven LEA protein family groups: LEA1-5, dehydrins, and SMPs (Table 1 and Supplementary Table S2). Hundertmark and Hincha [14] identified 51 LEAPs in model species *A. thaliana* and clustered them into nine groups: LEA1-5, dehydrins, seed maturation proteins (SMPs), PvLEA18, and AtM, although some lacked significant Pfam domains (as noticed with *R. serbica* LEAPs, particularly the LEA3 and LEA4 protein groups) and had high similarity to non-LEA protein families. Similarly, 242 LEAPs were identified in upland cotton, *G. hirsutum*, classified into eight groups ranging from LEA1 to LEA6, dehydrin, and SMP [15]. Forty identified wheat LEAPs were classified into six classes: LEApdB classes 1–4 containing the dehydrin domain (PF00257), LEApdB class 5 containing the PF00477 domain, and LEApdB class 6 containing the PF02987 domain [17]. Noticeably, the LEA6 protein family group was absent in *R. serbica*, as well as in *Oryza sativa* [19].

Interestingly, in a genome of a xerophyte perennial desert plant, *C. songorica*, only 44 putative LEA genes were identified and grouped into eight subfamilies, based on their conserved protein domains [23]. Similarly, in resurrection plants *C. plantagineum* and *D. hygrometricum*, only 16 and 21, respectively, were reported.

The initial analysis of the LEA genes in monocots and dicots revealed nearly half of them belong to the LEA4 and dehydrin families. The LEA4 group was the most dominant, followed by the dehydrins in Arabidopsis and the grapevine genome [14]. A special case presented LEAPs from *C. songorica*, which contained only one member of the LEA4 protein group [23]. In accordance with our data obtained for *R. serbica* LEAPs, the most abundant LEA protein group in tea plants was LEA2, encompassing ~40%, and LEA4, containing ~25%, of all LEAPs [18]. In agreement with that, the most populated LEA protein family group in upland cotton was LEA2 (encompassing 65% of all LEAPs compared with 40% in the case of *R. serbica*) [15]. A similar distribution of LEAPs was recently observed in *Sorghum bicolor*, where the most abundant group was LEA2 [21]. A possible reason for the smaller number of the LEA2 protein family group members described in the previously investigated genomes (such as poplar, rice, and Arabidopsis) might be the improvement of the higher plant genomes annotations and the gene duplication within this family group [18]. Indeed, in the recent comprehensive synteny and phylogenetic analyses of the eight LEA gene families (LEA1–6, SMPs, and dehydrins) across 60 complete plant genomes (not containing resurrection species), the LEA2 family was found as the most abundant, encompassing ~65% of all identified LEAPs, while LEA5 was a small family associated with 3.2% of all LEAPs, similar to that obtained in our study [2].

A phylogenetic analysis of *R. serbica* LEAPs showed that the LEA2 and LEA3.1 protein family (sub)groups were the last evolved *R. serbica* LEA families (Supplementary Figure S2). Considering the abundance and difference from other LEA family groups in cotton, Magwanga et al. [15] also suggested that LEA2 gene families might be the last evolved LEA gene family in higher plants. A recent thorough study on 458 LEAPs in 116 plant species revealed that the specific LEA3 protein motif arose early in land plant evolution [55]. On the other hand, a comprehensive study of 4863 LEAPs among 60 plant species proposed that the LEA5 group is the most conserved LEA protein family in plants [2]. The high number of paralogues, closely related genes exhibiting similar motif compositions, might be caused by whole-genome duplication and endoreplication events in the genome of *R. serbica*, a tertiary relict [21]. This emphasises the significance of the great diversity of the LEA proteome in plants that has been conserved during evolution [56].

### 3.2. Analysis of Amino Acid Composition and Physicochemical Properties of R. serbica LEAPs

The sequence length range of the identified *R. serbica* LEAPs was similar with that identified in bay beans [27] and tea plants [18]. However, in Arabidopsis [14] and cotton [15], bigger LEAPs were reported, reaching up to 67.2 to 160.7 kDa, respectively.

The range of pI values for *R. serbica* was following the one presented for the wheat LEAPs [17]. The average pI values obtained for the *R. serbica* LEA protein family groups showed better correlation with the *G. hirsutum* LEA groups, namely for dehydrins, SMPs, and LEA2 proteins, while neutral *R. serbica* LEA3 protein group members differed from significantly basic cotton LEA3 proteins [15].

The net hydrophobicity of each *R. serbica* LEA protein family group indicated that most LEAPs (except for some LEA2 protein group members) are hydrophilic in nature, as it was previously observed in other plants [14,15,17,53,57]. In agreement with the amino acid composition observed in *R. serbica* LEAPs, an exceptionally high content of lysine residues, particularly in dehydrins of *A. thaliana* and class 3 LEA (PF00257) in wheat, was reported [14,17]. Glycine was the most abundant amino acid in wheat LEA proteins [17], while its content was the highest in the *R. serbica* DEH1 and LEA5 protein family groups (Supplementary Table S2). The cysteine content was negligible in the *R. serbica* LEA1, LEA3, LEA4, LEA5, and DEH1 protein family (sub)groups, similar to in the most wheat LEA proteins, signifying that these proteins have a lower tendency to form disulphide bonds and fold into organised globular domains. This is in agreement with the previous analysis stating that LEA proteins lack or have a very low content of cysteine and tryptophan residues [53]. In agreement with *R. serbica* LEAPs, wheat LEAPs exhibited poor aromatic characters [17].

### 3.3. Protein Structure and Disorder Prediction of R. serbica LEA Proteins

Most LEAPs are predicted to be intrinsically disordered proteins (IDPs) [31,35]. The flexible structure of IDPs imposes restrictions on their 3D structure determination, as can be evidenced by a low number of deposed IDPs in the Protein Data Bank (PDB) [58]. Thus, an in silico analysis of the IDPs presents a valuable tool in their secondary structure evaluation.

This study employed five secondary structure predictors (including those specialised for IDPs, such as FELLS [59]) and four disorder estimators to evaluate and model the 3D structures of 318 identified *R. serbica* LEAPs (Supplementary Table S3). The results clearly underlined the differences among the annotated *R. serbica* LEA family groups (Figure 3). In proteins belonging to the LEA4 family group, the exceptionally high content of α-helices (particularly the so-called A type) was predicted. The random coil was the predominant secondary structure element in *R. serbica* dehydrins and LEA3.1 proteins. The significant content of β-sheets (37–58%) and lowest disorder propensity was assessed for *R. serbica* LEA2 protein group members. These findings were similar with the secondary structure prediction in bay bean LEAPs [27] and with wheat LEA proteins that mostly comprised the high helix and coil content and low β-sheet content, depending on the LEA class [17].

### 3.4. Subcellular Localisation of R. serbica LEA Proteins

Although the computational predictions of protein subcellular localisation provide important insights, high accuracy is not always achieved. Thus, the in silico results should be confirmed in vivo. Plant LEAPs are ubiquitously distributed over an array of intracellular components, including the cytoplasm [12], chloroplast [60], mitochondria [33], and nucleus [61,62]. Protection against the various adverse environmental conditions requires compartment-dependent stabilisation specific for different macromolecules, which is reflected by the redundancy and wide subcellular distribution of LEAPs.

### 3.5. Characterisation of the Individual R. serbica LEA Protein Family Groups and Estimation of Their Physiological Function under Desiccation

The induction of LEAPs is considered an essential part of the vegetative desiccation tolerance strategy in resurrection plants [3,63]. We aimed to propose the physiological functions of *R. serbica* LEAPs in desiccation tolerance, based on their structural properties and expression levels of the respective genes.

#### 3.5.1. *R. serbica* Dehydrins

In polylysine, the K-segment (KKGIMDKIKEKLPG) was found in many dehydrins [14,17,64,65], as well as in *R. serbica* dehydrins, particularly in the DEH1 subgroup (Supplementary Table S4). The K-segment appeared to be essential for binding to the anionic phospholipid vesicles [65] and was suggested to serve as a polar zipper to interact with DNA as well [61]. Related to that, a high lysine content found in the *R. serbica* DEH and LEA4 protein group members correlates very well with their predicted dominant nuclear localisation (Figure 6). Dehydrins identified in *R. serbica* are also rich in His residues, consistent with dehydrins from Arabidopsis [66] and *Citrus unshiu* [62]. Histidine residues interact via an imidazole ring with metal cations (Fe^+3^, Ni^+2^, and Cu^+2^) immobilised by negatively charged macromolecules [67,68]. Indeed, Zn^2+^ chelation by histidine residues was required for binding dehydrins to DNA [62].

Besides the K-segment, dehydrins contain Y- and S-segments (phosphorylation site) also used for their classification [21,23,69]. Both S- and Y-segments were observed in *R. serbica* dehydrins, especially in the DEH1 subgroup (Supplementary Table S4). The phosphorylated S-segment has been shown to cause dehydrin translocation from the cytoplasm to the nucleus [70] and also to increase the calcium-binding capacity of the protein [71].

Dehydrins identified in *R. serbica* showed the highest disorder propensity, particularly those belonging to the DEH1 subgroup (Supplementary Table S3). This is following previous reports, confirming that dehydrins can adopt various intrinsically disordered structures, making them quite dynamic in a solution [69]. However, in the presence of a membrane surface, dehydrins can gain a partial helical structure [72]. The representative RsLEA_139 (Figure 5) dehydrin was predicted to be highly disordered in the solution (97%), suggesting its possible involvement in liquid–liquid phase separation (LLPS), followed by proteinaceous condensates formation, similar to how it was recently experimentally confirmed for two Arabidopsis LEAPs predicted to be 100% disordered [35]. Therefore, nuclear desiccation-inducible RsLEA_139 might be involved in the LLPS-related dynamic assembly of nuclear compartments such as nuclear bodies and chromatin structures [73] as a part of gene expression regulation during desiccation in *R. serbica*. Temperature-dependent LLPS generation regulated the stress-related splicing activity of a fully disordered Ser and Arg-rich SR45 protein from Arabidopsis, allowing its accumulation in nuclear bodies [45].

In addition, all *R. serbica* DEH1 proteins plus seven DEH2 proteins were denoted as hydrophilins (Gly content > 6%; GRAVY index < 1, Figure 1) [74]. Six of the ten DEH1 gene members and one DEH2 were significantly upregulated upon desiccation in *R. serbica* leaves. It was reported that hydrophilins play a role in protecting cell components under osmotic stress [57]. However, definitive characterisation of their biochemical function(s) has remained somewhat elusive [69]. In vitro studies showed that dehydrins exhibit chaperone-like activity preventing protein aggregation, enzyme inactivation, and destabilisation of DNA and membranes upon heat exposure and freeze–thaw damage [37,75].

A hydroxyl radical (HO^•^) is involved in the oxidative modification/degradation of metabolites, proteins, lipids, and nucleic acids in plant cells, and its generation is accelerated during desiccation [10]. The hydroxyl radical scavenging ability (HRSA, Supplementary Materials Table S3), calculated based on the *R. serbica* dehydrin amino acid composition, was quite low. Keeping in mind that dehydrins were predicted to contain the highest percentage of random coil and, therefore, the highest molar fraction of solvent accessible residues, this might be expected in vitro. However, several in vitro studies have shown that dehydrins can protect lipids from oxidation by ROS and to reduce their generation in the presence of copper ions [75].

#### 3.5.2. *R. serbica* LEA1 Protein Family Group

The *R. serbica* LEA1 protein family members were characterised as very hydrophilic, highly disordered proteins. The sequences of this group display the unusual preponderance of glycine, lysine, and glutamate residues, similar to the same LEA protein family in Arabidopsis [14]. Members of this family group exhibited a high propensity to form amphipathic α-helices at the N-terminus and random coil at the C-terminus (Figure 5). This is in agreement with the already described structural properties, a variable C-terminal region, and a conserved portion at the N-terminal region predicted to form α-helices under water-limiting conditions [57]. For this reason, and their ability to accumulate in the plant cells in response to water stress, they are considered models to study IDPs in plants [31,40].

Interestingly, three proteins belonging to the *R. serbica* LEA1 protein family group (RsLEA_86, RsLEA_104, and RsLEA_263) exhibited a high similarity with two dehydration-inducible BhLEA proteins from *D. hygrometricum/B. hygrometrica* resurrection species [76]. The homologous segments: MQ[AT][VA]KQK[VM]S[ND][AS]AA[AST]AKE[HR]VD[VI][ML]KAKA[EQ] encompassed the M1.2 motif (Table 3) contained in 20 *R. serbica* LEA1 proteins. Tobacco plants overexpressing these genes were more tolerant to drought, as evidenced by more preserved proteins associated with photosynthesis and ROS scavenging, as well as by lower membrane permeability compared to wt plants. In addition, the above-mentioned segment was also detected in a dehydration-inducible LEA protein from the resurrection plant *C. plantagineum* [76].

In agreement with the above-mentioned study, half of the proteins belonging to the LEA1 family group were upregulated upon desiccation in *R. serbica* leaves (Table 4). Among them, five were distributed in mitochondria, five in the nucleus, and two in peroxisomes. The amphipathic α-helix allows LEA1 protein group members to stabilise cellular membranes by interacting with both nonpolar fatty acid tails and with phosphates of phospholipids (via lysine) as peripheral membrane-associated proteins. For example, the most upregulated RsLEA_86 protein (Figure 5), similar to drought-inducible BhLEAPs, might be involved in protecting the inner mitochondrial membrane. In Arabidopsis, a structural transition of a random conformation of LEA1 proteins in aqueous solutions, which turns into an α-helical structure under less water conditions, is suggested to be crucial in seed germination [34].

#### 3.5.3. *R. serbica* LEA2 Protein Family Group

On average, the largest and the bulkiest *R. serbica* LEAPs belonged to the LEA2 and LEA4 protein family groups. In *A. thaliana*, the biggest LEAPs were identified within the LEA4, AtM, dehydrins, and LEA2 protein family groups [14]. The LEA2 protein family members showed potential to fold into defined, globular domains, due to a high content of nonpolar amino acids and higher content of cysteine (1.4–2.0%), enabling the formation of disulphide bridges. These findings were confirmed in the study, encompassing 60 plant species [2]. Moreover, LEA2 family proteins are known to differ from other LEA proteins by high hydrophobicity, the existence of an atypical LEA domain known as the Water stress and Hypersensitive response (WHy) domain, and the highest level of the mean molar fraction of buried residues [74]. The WHy domain links NDR1/HIN1-like proteins (these domains were identified in some *R. serbica* LEA2 protein members by the InterPro database) involved in pathogen recognition to the Arabidopsis LEA14 protein (At1g01470) containing the PF03168–LEA2 member. However, members of the *R. serbica* LEA2 protein group showed a poor homology with the same group in Arabidopsis and upland cotton. The reason could lay in the observation that this protein family was found to be the most diverse LEA family in 60 plant species [2].

According to our HRSA calculations, *R. serbica* LEA2 proteins were annotated as the most potent hydroxyl radical scavengers (Supplementary Table S3). Physiological functions of the LEA2 protein family group members are associated with salinity, freezing, heat, UV radiation, osmotic, and oxidative stress in vitro [77].

Most of *R. serbica* LEA2 protein members (LEA2.3–2.5) protein subgroups contained disordered N-terminal regions, followed by transmembrane hydrophobic α-helices (TMH) and a compact globular domain in the form of β-barrel at the C-terminus (Figure 5 and Figure S11). They are distributed in many subcellular compartments, while those containing the TMH 2.3–2.5 subgroup accumulated preferentially in chloroplasts (Figure 6). The latter might be located within the thylakoids and protect these particularly important photosynthetic components during water scarcity. Moreover, highly abundant arginine residues might additionally interact with the negatively charged phospholipids, similar to how it was reported for LEA2 proteins and anionic phospholipid vesicles [65]. The LEA2.2 protein group members form a single amphipathic α-helix that can interact with fatty acid chains in chloroplastic membranes and stabilise them. However, upon desiccation, only six out of 88 members of LEA2.3–LEA2.5 subgroups were upregulated (four chloroplastic, one extracellular, and one vacuolar protein), while 16 were downregulated (seven chloroplastic proteins). These results indicated the possible involvement of other LEA protein family groups (LEA4 and/or SMP) in the protection of the chloroplastic membranes.

Taken together, *R. serbica* LEA2 should be regarded as an unusual protein family group composed of a higher portion of hydrophobic amino acids, with a more defined secondary structure in the solution compared with the other LEA families.

#### 3.5.4. *R. serbica* LEA3 Protein Family Group

The LEA3 protein group family was the second smallest *R. serbica* LEA protein family, encompassing 18 quite short (103–156 aa) members, following the LEA5 group, which is in a good correlation with the LEA3 proteins from other plant species [55]. *R. serbica* LEA3 proteins had an averaged GRAVY index of –0.59, which, despite being negative, was higher (i.e., less polar) than the other LEA groups that tended to group around –1.2 (except LEA2 and SMP). The most interesting feature of these proteins was their high tryptophan and proline contents, particularly in the LEA3.2 subgroup. Tryptophan was preserved entirely in the M3.1 and M3.5 motifs, characteristic for the LEA3.1 and LEA3.2 protein members, respectively (Figure 2 and Figure S6). The so-called W-motif: W[VMTA]P[DH][PE][VKR]TG[YIGF][YWF][RYFT]P[EKA][NGT], found in 458 LEAPs belonging to 116 plant species (*D. hygrometricum* was not included) [55], corresponds very well with the fully conserved sequence: WAPHPKTGVFGPA, part of the *R. serbica* M3.1 motif (Table 3). In the case of the representative RsLEA_202 protein, the M3.1 motif formed an α-helix located closer to its C-terminus (Figure 5), similar to in the LEA3.2 subgroup presented in Reference [55]. The same comprehensive study also detected the RRGYA_4_ motif denoted as M3.5 and DAAELR segment identified in the M3.4 motif (Table 3).

The in silico analysis of proteins belonging to the *R. serbica* LEA3 family group showed that they should be mainly distributed in mitochondria and chloroplasts. This is in accordance with the prevalent localisation of *A. thaliana* LEA3 family members [56]. Moreover, the RRGYA_4_ motif can serve as a signal for the localisation of the LEA3 protein family members into mitochondrion [55]. This also correlates well with the secondary structure prediction, since plant mitochondrial-directing peptides typically possess an amino acids sequence with a propensity to form an α-helix. The preliminary biophysical results suggested that the *A. thaliana* LEA3 proteins are disordered in the solution [55], which fits very well with the results obtained for the *R. serbica* LEA3 protein family group, particularly the LEA3.2 subgroup. Upon desiccation, only one LEA3 gene family member-encoding protein located in chloroplasts was overexpressed (Table 4). In contrast, three LEA3 genes were significantly downregulated in *R. serbica* DL, particularly RsLEA_128, predicted to be located in the mitochondria. Although the (over)expressed *A. thaliana* LEA3 protein member improved the oxidative stress and drought tolerance (e.g., against H_2_O_2_) in yeast and transgenic plants [25], the calculated HRSA for the *R. serbica* LEA3 protein group was quite low. In addition, the maize protein LEA3 group has been able to stabilise the membranes and proteins during low-temperature exposure, osmotic stresses, and against H_2_O_2_ [26].

#### 3.5.5. *R. serbica* LEA4 Protein Family Group

The most striking features of the *R. serbica* LEA4 protein family members were their hydrophilic characters and a high percentage of lysine, glutamate, and aspartate, as well as the significantly high propensity for adopting the α-helical structure (Figure 4 and Figure 6). At first glance, unexpectedly high contents of hydrophobic alanine found in the generally hydrophilic LEA4 protein family group, particularly in LEA4.3 (18%), can be correlated with the extremely high helical content in this group. Alanine was identified as a former α-helix [78]. The same situation was observed in wheat class 6 LEAPs (PF02987, analogous to *R. serbica* LEA4) [17]. Moreover, motifs corresponding to the *R. serbica* LEA4 protein family group folded into so-called A-type α-helices (also present in the M4.3 and M4.4 motifs) that contained positive, negative, and hydrophobic faces (Figure 5). Similarly, during dehydration, two mitochondrial LEAPs from peas folded into an amphipathic helical form, the A-type α-helix, allowing them to immerse laterally within the inner layer of the inner membrane, reinforcing the membrane in the dry state [31,32,33,36]. In addition, all *A. thaliana* LEA4 protein members harboured the class A α-helix motifs [56].

As a confirmation of the results calculated for the *R. serbica* LEA4 protein family group members, both the experimental and prediction data indicated that members of the LEA4 protein family group were distributed in several cellular compartments [56]. Therefore, a hydrophobic strip on the class A α-helices might be orientated towards the fatty acid tails of the outer plasma membrane (in the case of extracellular *R. serbica* LEA4 proteins) or inner mitochondrial or peroxisomal membranes (mitochondrial and peroxisomal *R. serbica* LEA4 proteins), while the positive strip on these helices can form electrostatic interactions with negatively charged phosphate groups of phospholipids. In this way, as peripheral membrane-associated proteins, they would provide support for the membranes, as shown in vitro with class A α-helix-containing Arabidopsis LEA4 proteins [36]. The lipid composition of the inner envelope membrane of the chloroplasts, etioplasts, or proplastids and thylakoids comprise a high proportion of neutral galactolipids and only 8–10% phospholipid [56]. This could play a role in the stress protection of thylakoids, although the electrostatic interactions between the A-type α-helical domains of the LEA4 proteins could not be obtained. Therefore, it is more likely that desiccation-induced chloroplastic RsLEA_301, a LEA4 member, via its A-type of the α-helix composed of positive and negative sides, can interact with desiccation-sensitive proteins in chloroplasts, particularly photosynthetic electron transport components. At the same time, this protein was annotated as a highly disordered protein (Supplementary Table S3). Therefore, RsLEA_301 (and similar LEA4 members) might adopt a random coil in aqueous solution and fold into an α-helix when subjected to water deficit and/or macromolecular crowding environments. Indeed, the LEA4 proteins from *A. thaliana* showed the ability to gain an α-helical structure under water-limiting conditions to prevent the inactivation and/or aggregation of lactate dehydrogenase, the reporter enzyme in vitro [31]. This corresponds with the structural plasticity of IDPs able to select one of their fluctuating conformations, which can further be locked by the contact with their partner protein.

In the case of the nuclear LEA4 proteins, such as upregulated RsLEA_188 gathering positive residues to form a negatively charged strip of the A type of the α-helix (according to the HeliQuest webserver) almost along the whole sequence length (~95, Supplementary Materials Table 3) can be important for binding and stabilising DNA.

#### 3.5.6. *R. serbica* LEA5 Protein Family Group

As obtained for the wheat LEA family group containing the LEA5 domain (PF00477) [17], the smallest *R. serbica* LEAPs belonged to the least-populated LEA5 protein family group. In agreement with that, the LEA family group with the least members in *G*. *hirsutum* was LEA5, containing 3.7% of the LEAPs [15]. In addition, the smallest cotton LEAPs (average) belonged to the LEA3 and LEA5 protein family groups [15]. Similarly, the smallest *A. thaliana* LEAPs generally belonged to the PvLEA18 and LEA5 protein family groups [14]. Contrary to the results regarding wheat LEA5 proteins, calculated to be acidic, the *R. serbica* LEA5 protein group members were basic (pI = 8.1).

The genes belonging to the LEA5 group were the most upregulated upon desiccation among all 318 *R. serbica* LEA genes. Desiccation increased the expression levels of seven of the eleven genes encoding LEA5 proteins, while five of them were predicted to accumulate in the nucleus. The representative nuclear RsLEA_202, mostly composed of α-helices and random coils, was the highest induced LEAP in *R. serbica* DL.

#### 3.5.7. *R. serbica* SMPs

The seed maturation protein family group was the most acidic *R. serbica* LEA group (Table 2), similar to the SMPs detected in sorghum (pI = 4.8) [21]. It was proposed that the SMP family group arose early in the plant lineage (together with the LEA5 family), while the other families appeared at later instants during plant evolution [2]. This might have a great influence during the colonisation of the terrestrial environments by embryophytes. The seed maturation protein family was also detected in desiccation-tolerant brine shrimp *Artemia franciscana* [28].

The in silico analysis of *R. serbica* SMPs showed that they should be mainly distributed in chloroplasts but also in the cytosol and nuclei (Figure 6).

Interestingly, a recent in vivo study evidenced that an SMP domain (PF04927) of the AfLEA6 protein promoted LLPS in vivo and formed the condensates that contributed to the desiccation tolerance in *A. franciscana* by increasing the cytoplasmic viscosity and by providing protective compartments for desiccation-sensitive proteins [28]. AfLEA6 contains the M7.1 motif identified in the members of the *R. serbica* SMP2 and SMP3 subgroups (Figure 2). Seven chloroplastic (six SMP2) and three cytosolic SMP members were upregulated in DL compared with HL of *R. serbica*. The representative RsLEA_71 cytosolic desiccation-induced protein belonging to the SMP3 subgroup contained the M7.1 and M7.2 motifs, which can adopt the structure of the amphipathic α-helix. Thus, this protein may endorse desiccation tolerance in two ways, by LLPS and proteinaceous condensate-building (as observed for AfLEA6) and by direct physical and functional interactions with the membranes delimiting the organelles protecting cytosol and organelles as well. The protective role of SMPs against salt stress was evidenced in the case of bay beans [27].

Our comprehensive in silico and gene expression pattern analyses stressed structural, physicochemical, localisation, and biological differences between seven LEA protein family groups in *R. serbica* HL and DL. Compared with LEAPs belonging to desiccation-sensitive plant species, an exceptionally high number (318) of identified LEAPs indicate that they confer an evolutionary advantage for this ancient resurrection plant species to cope with extremely adverse environmental conditions such as desiccation. On the other hand, a relatively small number of LEAPs was reported for desiccation-tolerant *C. plantagineum* and *D. hygrometricum*. Our in silico findings will be experimentally validated in our further studies. Nevertheless, the presented study is an important starting point for future efforts to elucidate the mechanism of action at the cellular level and biochemical characterisation, especially their large structural flexibility, which is still lacking.

## 4. Materials and Methods

### 4.1. Plant Material and Treatment

The resurrection plants *Ramonda serbica* Pančić were collected from their natural habitat in a gorge near the city of Niš in South-eastern Serbia. Desiccation was induced as described previously in Reference [46].

### 4.2. De Novo Transcriptome Analysis of R. serbica HL and DL

#### 4.2.1. RNA Extraction, cDNA Library Construction, and Illumina High-Throughput Sequencing

For *R. serbica* transcriptome construction, high-quality RNA from HL and DL (mix of four plants, three leaves per plant) were extracted according to our previously optimised TRIzol-based protocol [5]. The total RNA quality and quantity assessment and cDNA library construction using the Illumina HiSeq 4000 platform (Illumina, Inc., San Diego, CA, USA) and quality evaluation on the Agilent Bioanalyzer 2100 system were recently described in detail [5]. Clustering of the index-coded samples was performed on a cBot Cluster Generation System using PE Cluster Kit cBot-HS (Illumina) according to the manufacturer’s instructions.

#### 4.2.2. Transcriptome De Novo Assembly and Sequence Annotation

The raw reads from Illumina were transformed to sequenced reads by base calling (in FASTQ format). The obtained reads were processed through in-house scripts to remove reads containing adapter sequences, poly-N sequences, and reads with low quality. The clean data’s Q20, Q30, and GC contents were calculated. The obtained high-quality clean reads were subjected to de novo assembly using Trinity [79]. The redundancies from the Trinity results were removed by the Corset method [80]. The longest transcripts of each cluster (Corset-filtered contigs) were selected as unigenes. Hierarchical clustering was performed based on multiple mapping events and expression patterns.

Functional annotation of the unique assembled transcripts was performed using the following databases: NCBI nonredundant (NR) protein sequences, NCBI NR nucleotide sequences, the Protein family (Pfam) database [81], Clusters of Orthologous Groups of proteins (KOG/COG), Swiss-Prot, the Kyoto Encyclopedia of Genes and Genome (KEGG) Ortholog database [82], and Gene Ontology, GO, by the GOseq R package.

#### 4.2.3. Differential Expression Analysis and Functional Enrichment

To identify differentially expressed genes (DEGs) between *R. serbica* HL and DL, the expression level of each transcript was calculated according to the FPKM method. The gene expression levels were estimated by RSEM [83] for each sample: (i) the clean data were mapped back onto the assembled transcriptome, and the (ii) read count for each gene was obtained from the mapping results. Prior to the DEG analysis, the read counts were adjusted by the edgeR program through one scaling normalised factor for each sequenced library. A DEG analysis between HL and DL was performed using the DEGseq R package. The *p*-values were adjusted using the Benjamini and Hochberg methods. A corrected *p*-value of 0.005 and |log2^(Fold Change)^| of 2 were set as the threshold for significantly differential expression.

A functional enrichment analysis, including GO and KEGG terms compared to the whole-transcriptome background, was performed. A GO enrichment analysis of differentially expressed genes was implemented by the GOseq R package, in which the gene length bias was corrected. GO terms with corrected *p*-value < 0.05 were considered significantly enriched by differential expressed genes. Statistical enrichment of differential expression genes in KEGG pathways was tested using KOBAS software [84].

### 4.3. Identification and Classification of R. serbica LEAPs

*Ramonda serbica* LEA sequences were searched against the NCBI NR protein database by using the Basic Local Alignment Search Tool (BLAST [85]). The search space was reduced down to the taxonomy id for land plants (3193). Only full-length sequences with an e-value cut-off of 10^−6^ and sequence identity > 90% were considered for inclusion in the *R. serbica* LEAPs list, and the annotations were checked manually. A unique *R. serbica* LEAP list was generated by manually sorting annotated LEAPs using the Pfam [81], InterPro [86], and Panther databases [87] to verify the presence of the LEA protein domains. To obtain functional domain information from Pfam, HMMPfam was run with an e-value threshold of 10^−5^. For the annotation of the LEA2 protein family group, Phyre2 annotation (high confidence and alignment coverage for NMR resolved structures of two At2g46140.1 and At1g01470 LEAPs) was taken into account as well. Finally, proteins consisting of less than 100 amino acids (aa) were omitted.

### 4.4. Physiochemical Characterisation of R. serbica LEAPs

The physicochemical characterisations of the *R. serbica* LEAPs were done by computing the sequence length, isoelectric point (pI), amino acid composition, protein’s molecular weight, with the Expasy’s ProtParam server (http://web.expasy.org/compute_pi/, accessed on 8 February 2022). Using BioPython (v1.77), the amino acid-based properties were computed for each sequence (see Supplementary Table S2) [88]. The evaluation of the grand average of hydropathicity (GRAVY), a measure of a protein’s hydrophobicity and solubility, of the identified LEAPs was performed by the GRAVY calculator (http://www.gravy-calculator.de/, accessed on 8 February 2022). A negative GRAVY value denotes a hydrophilic protein, while a positive value denotes that the protein is hydrophobic. In order to reveal hydrophylin-type proteins (GRAVY < 1 and Gly > 6%, [2,74]), individual GRAVY scores were plotted against the percentage of Gly per protein sequence. The same plots were built for Lys+Glu, Ala, Ile+Leu+Val, Cys, Trp, His, and Pro percentages.

### 4.5. Phylogenetic Identification of R. serbica LEAPs

A phylogenetic tree was constructed to understand the evolutionary relatedness among *R. serbica* LEAPs. Multiple sequence alignment (MSA) of the full-length sequences of *R. serbica* LEA proteins was performed using the MAFFT v7 [89] L-INS-i method with 1000 iterations of improvement, the BLOSUM62 scoring matrix, and gap opening penalty of 1.53. A phylogenetic tree was created using the EMBL-EBI Simple Phylogeny tool [90] with the neighbour-joining method and default parameters. iTOL–Interactive Tree Of Life v.6.5 [91] was used to display and annotate the tree.

Homology comparison of the members of the specific *R. serbica* LEA protein family groups with the members of the corresponding LEA groups found in *A. thaliana* and *G. hirsutum* [14,15] was performed using the MAFFT tool with an autodetected alignment algorithm comparing the pairwise sequence alignment. Finally, the average sequence identity within the specific LEA protein family group of the two species was compared.

### 4.6. Conserved Motif Composition in R. serbica LEAPs

The Multiple Expectation Maximization for Motif EliCitation (MEME) online tool [92] was used to identify the conserved protein motifs. MEME was run using the “zero or one occurrence per sequence” mode and searched for 3–15 different motifs (depending on the LEA protein family group) with a minimum width value of 6 and a maximum width of 50. All other parameters were left at their default values. The obtained MEME outputs (in XML format) were exported into interactive iTOL online software [91] to couple and visualise the motifs with the phylogenetic tree of each LEA protein family group separately.

### 4.7. Secondary Structure and Disorder Predictions of R. serbica LEAPs

The secondary structure estimation of the *R. serbica* LEAPs was performed using the following predictors: (i) Sopma [93], (ii) PsiPred [94], (iii) Phyre2 [95], (iv) FELLS [59], and (v) JPred4 [96].

The prediction of the possible transmembrane α-helices (TMH) in the identified LEAPs was obtained with the TMHMM predictor [97]. The mean hydrophobicities and amphipathicities of the predicted TMH were calculated with the analysis procedure on the HeliQuest webserver [47]. The amino acid distribution and amphipathicity assessment for TMH predicted in LEAPs were projected in helical wheel diagrams.

The disorder estimation was performed via specialised disorder predictors: the FELLS [59], IUPred3 [98], and ESpritz sequence-based methods for disorder determination built on machine learning and bidirectional recurrent neural networks. Two methods based on ESpritz disorder prediction were employed. The first was Espritz-DisProt, based on the MSA of the target LEAP and proteins deposited in the DisProt database [99]. DisProt is a manually curated database of partially or completely disordered proteins [100]. The second method was Espritz-X, which relies on the crystal structures obtained by X-ray crystallography from the PDB database [101], where residues lacking coordinates for any of the backbone αC atoms are denoted as disordered.

### 4.8. Modelling 3D Protein Structure

LEAPs sequences were used as an input for Colabfold [102]. The protein structure was predicted using AlfaFold2 [103]. The 3D protein structure was visualised in PyMOL v.2 (https://pymol.org/2/, 9 February 2022).

### 4.9. Annotation of the Subcellular Localisation of R. serbica LEAPs

In addition, the subcellular location prediction of LEAPs was conducted using the TargetP1.1 server [104] and Protein Prowler Subcellular Localisation Predictor version 1.2 [105]. To predict the specific compartmentalisation of each LEAP, the WoLF-PSORT tool was used [106].

### 4.10. Statistics

Tukey’s post hoc test was used to test for significant differences in the calculated protein parameters among the different LEA protein family groups by IBM SPSS statistics software (v20.0, IBM Corp., Armonk, NY, USA). The significance threshold value was set at 0.05. The standard error of the mean in the species similarity comparison was calculated using the SciPy statistics module [107].

## 5. Conclusions

This study presents the first comprehensive structure–function characterisation of LEAPs in a relict endemic resurrection plant *Ramonda serbica*. In total, 318 LEAPs from hydrated and desiccated leaves were identified and classified into the seven LEA protein family groups ranging from LEA1-LEA5 and SMPs to dehydrins. An analysis of the physicochemical properties, motif architecture, secondary structure, homology, and phylogenetic relationships demonstrated that *R. serbica* LEAPs greatly differed among the LEA family groups. Proteins belonging to the most abundant group, LEA2, were atypical due to their lower hydrophilicity and high propensity to fold into organised globular domains with a conserved transmembrane α-helix. The genes encoding the LEA2 proteins presented the majority downregulated by desiccation. On the other hand, the LEA4 proteins were highly hydrophilic, desiccation-induced, and widely distributed in the cells. They exhibited an exceptionally high propensity to form A-type α-helical structures with differentiated charged and hydrophobic faces. Desiccation-upregulated nucleolar dehydrins are rich in histidine and lysine residues, required for metal chelation and DNA binding. Additionally, a group of desiccation-upregulated *R. serbica* LEAPs, particularly dehydrins (hydrophilins), LEA1, and LEA3 proteins, are recognised as highly disordered proteins. As such, they are able to promote LLPS-driven condensate forming and endorse desiccation tolerance by increasing the cytoplasmic and stromal viscosity, as well as by providing protective compartments for desiccation-sensitive proteins. Moreover, turning from a random conformation into the (amphipathic) α-helices during dehydration enabled them to stabilise various partners (e.g., membranes and target proteins) in different cellular compartments. Taken together, possible functions of LEAPs are proposed with significant implications on the drought tolerance improvement of crops grown in arid areas.

## Figures and Tables

**Table 1:** Sequence similarity of *R. serbica* LEAPs with LEAPs from *A. thaliana* and upland cotton, *G. hirsutum*.

LEA Protein Family Group	Pfam ID	Protein Number	*A. thaliana* Similarity, %	*G. hirsutum* Similarity, %
LEA1	PF03760	24	41.5 ± 0.9	28.8 ± 0.4
LEA2	PF03168	127	32.8 ± 0.9	29.8 ± 0.3
LEA3	PF03242	18	34.7 ± 1.6	27.7 ± 0.5
LEA4	PF02987	96	29.8 ± 0.3	28.2 ± 0.3
LEA5	PF00477	11	58.6 ± 4.4	27.8 ± 0.5
Dehydrin	PF04927	25	41.6 ± 1.6	34.4 ± 0.9
SMP	PF00257	17	37.9 ± 0.9	24.7 ± 0.3

Values represent mean ± SE.

**Table 2:** Sequence-dependent characteristics of LEAPs from *R. serbica*. The physicochemical parameters of each LEAP were calculated by using the ExPASy online server (http://web.expasy.org/compute_pi/, accessed on 8 February 2022) and by the GRAVY calculator (http://www.gravy-calculator.de/, accessed on 8 February 2022).

LEA Protein Group	aa #	Calculated pI	Mw (kDa)	GRAVY Index	Amino Acid (aa) Composition				
					%	%	%	%	%
					Charged	Polar	Nonpolar	Aromatic	Cys
LEA1	139 ± 5	8.2 ± 0.4 ^d^	14.4 ± 0.5 ^a^	−0.93 ± 0.05 ^b^	23.2 ± 1.3 ^a^	33.2 ± 0.9 ^c,d^	40.5 ± 0.8 ^c^	5.1 ± 0.5 ^a^	0.10 ± 0.06 ^a^
LEA2	226 ± 5	8.4 ± 0.3 ^d^	25.2 ± 0.7 ^b^	−0.09 ± 0.03 ^e^	21.4 ± 0.5 ^a^	29.3 ± 0.5 ^b,c^	38.8 ± 0.4 ^b,c^	9.7 ± 0.2 ^c^	1.66 ± 0.10 ^c^
LEA3	126 ± 5	7.0 ± 0.4 ^b,c,d^	14.0 ± 0.6 ^a^	−0.59 ± 0.03 ^c^	23.6 ± 0.5 ^a^	35.6 ± 1.0 ^d^	32.3 ± 0.8 ^a^	10.5 ± 0.4 ^c^	0.56 ± 0.09 ^a,b^
LEA4	187 ± 7	6.1 ± 0.2 ^a,b^	17.9 ± 0.9 ^a^	−1.01 ± 0.03 ^b^	35.2 ± 0.6 ^b,c^	23.6 ± 0.5 ^a^	36.7 ± 0.5 ^b^	3.7 ± 0.2 ^a^	0.33 ± 0.06 ^a,b^
LEA5	118 ± 7	8.1 ± 0.5 ^c,d^	12.7 ± 0.8 ^a^	−1.02 ± 0.14 ^b^	30.0 ± 1.4 ^b^	29.2 ± 1.7 ^b,c^	36.9 ± 1.7 ^b,c^	4.7 ± 0.9 ^a^	0.56 ± 0.28 ^a,b^
Dehydrin	143 ± 9	6.7 ± 0.5 ^b,c^	15.6 ± 1.0 ^a^	−1.40 ± 0.05 ^a^	37.2 ± 2.7 ^c^	28.7 ± 1.5 ^b,c^	29.3 ± 1.5 ^a^	9.5 ± 0.5 ^c^	1.00 ± 0.19 ^b,c^
SMP	157 ± 15	4.9 ± 0.3 ^a^	16.4 ± 1.6 ^a^	−0.27 ± 0.04 ^d^	22.9 ± 0.8 ^a^	26.9 ± 1.3 ^a,b^	46.6 ± 1.0 ^d^	6.8 ± 0.4 ^b^	0.88 ± 0.21 ^b^

Values represent the mean ± SE; different letters denote statistically significant differences between different LEA protein family groups (*p* < 0.05).

**Table 3:** A consensus sequence of the different motifs features of each R. serbica LEA protein family.

Protein Family	Motif	aa no.	Motif e-Value	Consensus Sequence	Gravy Index	Consensus Logo *
LEA1	M1.1	50	1.25 × 10^−52^	TKATVQEKAEQMKTRDPLQKEMATQKKEAKIQEAERQKQEARQQNSAAKH	−1.786	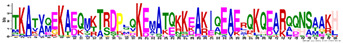
	M1.2	21	3.8 × 10^−21^	MQAAKETAANVAASANSGMEK	−0.352	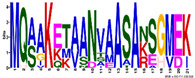
LEA2	M2.1	50	5.4 × 10^−55^	IEETIGFGKPTADVTDVDLKDINLEKADYVVDVLVKNPYPIPIPLIDINY	0.048	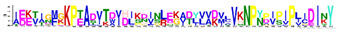
	M2.2	50	2.7 × 10^−55^	KSTYADIGPGWIIPYRLKVDLIVDVPVFGRLTLPLEKKGEIPIPYKPDID	−0.018	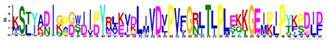
	M2.3	50	5.1 × 10^−63^	IRFDKFSFEETVATLHLKLENKNDFDLGLKDLDYEVWLCNVSIGGAYMKK	−0.268	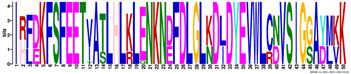
	M2.4	50	1.1 × 10^−44^	TLNLTVTVRNPNFYSIKYDSSTVSIGYRGNKLGRVTIPAGRIGARSSQRV	−0.328	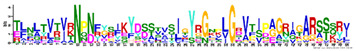
	M2.5	50	1.9 × 10^−64^	ITFRPKDFGSALWDMIRGKGTGYTIKGNINVDTPFGFMKLPISKEGGTTC	−0.238	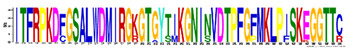
	M2.6	29	1.9 × 10^−34^	SGLIPDAGSLKAHGSTTVKVPICLIYDDI	0.444	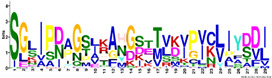
	M2.7	50	3.1 × 10^−57^	NATLQLERVEIMSDVILLLEDLAKGEIMFDTEVDISGKLRVFFFDLPLKT	0.376	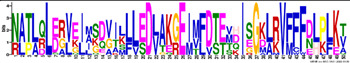
	M2.8	21	1.6 × 10^−22^	RNPNKRIGIYYDQIDAYASYK	−1.200	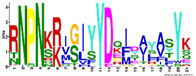
	M2.9	24	1.2e^−28^	GGGKRINDKGWPECNVIMEEGKYD	−1.204	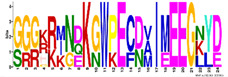
LEA3	M3.1	41	1.8 × 10^−53^	TYDKNPDEEHAFSAVVPDNVIPPQTQQYWAPHPKTGVFGPA	−0.817	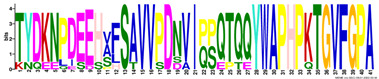
	M3.2	29	1.6 × 10^−36^	SVSNGGADSVLEQKAFFRPLEDLDKPHHP	−0.766	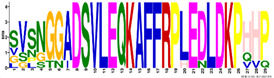
	M3.3	29	2.4 × 10^−35^	MAANLQSRGLASFSKQFVIRVRSRDSTII	0.048	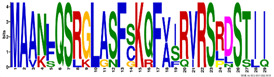
	M3.4	50	1.9 × 10^−48^	IRMLNKESEEPTKISWVPDPVTGYYRPENKATEIDAAELRRILIKDNTRR	−0.994	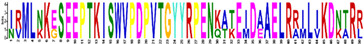
	M3.5	6	1 × 10^−8^	RRGVHV	−0.700	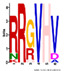
LEA4	M4.1	29	8.5 × 10^−29^	AKDYVADKAKEAKDSAAEKAKETKDKAGE	−1.617	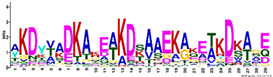
	M4.2	29		IIGSLIGTVQGTVEHAKEAVLGKSQEASE	0.059	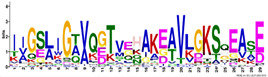
	M4.3	36		AKMKAEDTTEAAKETYEETKENARKKMEEMKIVGEG	−1.962	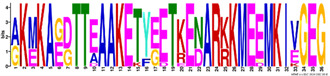
	M4.4	21		AKEKAKEAKDSAKDKAGETKD	−1.438	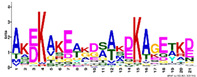
LEA5	M5.1	50	1.8 × 10^−60^	QDKRAELDAKASQGETVVPGGTGGKSLEAQEHLAEGRSKGGQTRKEQMGT	−1.228	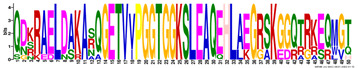
	M5.2	21	3.4 × 10^−36^	YQEMGRKGGLSSNDKSGAERAEEEGITID	−1.256	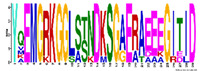
Dehydrins	M6.1	29	2.3 × 10^−30^	GGGGVAGQEEPEKKGMMDKIKEKLPGGHH	−1.214	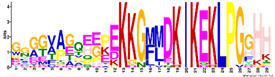
	M6.2	29	8.4 × 10^−33^	GPTTGPPKHRRSGSSSSSSSEDDGMGGRR	−1.679	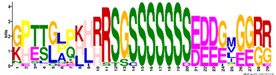
	M6.3	29	7.2 × 10^−37^	MAEYGGNYGNETKQTDEYGNPVHHPQGGG	−1.559	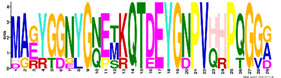
	M6.4	21	5.4 × 10^−23^	KGLKDKIKEKLPGGKKETEPP	−1.710	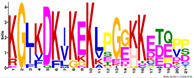
SMP	M7.1	50	3.5 × 10^−60^	PQDAATMQAAENSVLGQTQKGGVAATMQSAANRNERAGVVGHNDVTDIIS	−0.402	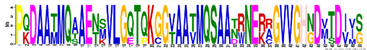
	M7.2	41	1.3 × 10^−48^	SAAGDKPVDESDAAAIQAAEARATGLGRVVPGGLGAEAKSA	−0.090	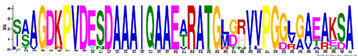

* Different-sized letters in the MEME sequence logos denote the individual residue probabilities. Important motif components are bolded. The colour scheme of the logo indicates the amino acid types: polar uncharged, green; positively charged, red; negatively charged, pink; nonpolar, blue; Gly, orange, Pro, yellow; His, light pink; Tyr, cyan.

**Table 4:** Differentially expressed genes encoding annotated LEAPs in *R. serbica* upon desiccation.

Subgroup	Rs_id	LEAP_id	log2(DL/HL)	Subgroup	Rs_id	LEA_id	log2(DL/HL)
LEA1.1	Rs_164046	RsLEA86	6.97	LEA3.1	Rs_161911	RsLEA85	−3.48
LEA1.1	Rs_152347	RsLEA78	6.39	LEA3.2	Rs_114021	RsLEA128	−9.07
LEA1.1	Rs_185287	RsLEA104	5.24	LEA4.2	Rs_146887	RsLEA75	6.42
LEA1.1	Rs_186228	RsLEA277	3.91	LEA4.2	Rs_131921	RsLEA312	5.87
LEA1.1	Rs_116928	RsLEA44	3.74	LEA4.2	Rs_194183	RsLEA188	4.99
LEA1.1	Rs_105968	RsLEA146	2.52	LEA4.2	Rs_186681	RsLEA310	4.94
LEA1.1	Rs_125102	RsLEA52	2.45	LEA4.2	Rs_148951	RsLEA76	3.10
LEA1.1	Rs_172584	RsLEA267	2.43	LEA4.2	Rs_146172	RsLEA316	−4.35
LEA1.1	Rs_183967	RsLEA101	2.35	LEA4.2	Rs_182435	RsLEA51	−4.91
LEA1.1	Rs_156613	RsLEA266	2.21	LEA4.3	Rs_190897	RsLEA110	6.04
LEA1.3	Rs_170082	RsLEA129	3.00	LEA4.3	Rs_189187	RsLEA109	5.64
LEA1.3	Rs_108065	RsLEA26	2.55	LEA4.3	Rs_131918	RsLEA311	5.12
LEA2.0	Rs_130914	RsLEA122	−2.24	LEA4.3	Rs_109487	RsLEA175	4.90
LEA2.1	Rs_169359	RsLEA232	2.93	LEA4.3	Rs_109602	RsLEA301	4.29
LEA2.1	Rs_127322	RsLEA55	2.33	LEA4.3	Rs_184475	RsLEA309	3.77
LEA2.2	Rs_151841	RsLEA154	4.64	LEA4.3	Rs_149505	RsLEA130	2.95
LEA2.2	Rs_104785	RsLEA275	4.51	LEA4.3	Rs_181059	RsLEA302	2.74
LEA2.2	Rs_125141	RsLEA276	3.66	LEA4.3	Rs_136891	RsLEA314	2.73
LEA2.2	Rs_164865	RsLEA272	2.50	LEA4.3	Rs_190898	RsLEA111	2.31
LEA2.2	Rs_187807	RsLEA125	2.07	LEA4.3	Rs_108999	RsLEA36	2.18
LEA2.2	Rs_173883	RsLEA265	−2.98	LEA4.3	Rs_166537	RsLEA49	1.99
LEA2.2	Rs_166384	RsLEA269	−4.06	LEA4.3	Rs_172003	RsLEA95	−3.63
LEA2.3	Rs_194495	RsLEA270	3.77	LEA5	Rs_188268	RsLEA202	11.80
LEA2.3	Rs_110370	RsLEA262	3.19	LEA5	Rs_159833	RsLEA196	8.53
LEA2.3	Rs_121097	RsLEA261	3.09	LEA5	Rs_128109	RsLEA200	8.13
LEA2.3	Rs_118201	RsLEA230	2.44	LEA5	Rs_193475	RsLEA204	8.04
LEA2.3	Rs_183071	RsLEA264	2.39	LEA5	Rs_124807	RsLEA201	5.55
LEA2.3	Rs_193485	RsLEA291	−2.14	LEA5	Rs_125649	RsLEA199	4.35
LEA2.3	Rs_171129	RsLEA256	−2.59	LEA5	Rs_176248	RsLEA203	2.86
LEA2.3	Rs_138912	RsLEA296	−2.71	DEH1	Rs_131408	RsLEA166	3.78
LEA2.3	Rs_145248	RsLEA285	−2.89	DEH1	Rs_172145	RsLEA139	3.38
LEA2.3	Rs_180651	RsLEA98	−3.46	DEH1	Rs_134636	RsLEA298	3.09
LEA2.3	Rs_138298	RsLEA68	−5.55	DEH1	Rs_107019	RsLEA152	2.78
LEA2.4	Rs_110833	RsLEA254	−2.55	DEH1	Rs_181340	RsLEA151	2.50
LEA2.4	Rs_181906	RsLEA257	−3.77	DEH1	Rs_113392	RsLEA163	2.30
LEA2.5	Rs_160078	RsLEA228	8.12	DEH2	Rs_156753	RsLEA172	4.35
LEA2.5	Rs_159852	RsLEA239	3.66	SMP1	Rs_140935	RsLEA70	7.78
LEA2.5	Rs_162712	RsLEA211	3.34	SMP1	Rs_106521	RsLEA33	3.67
LEA2.5	Rs_139255	RsLEA244	−2.32	SMP2	Rs_135719	RsLEA66	8.03
LEA2.5	Rs_186090	RsLEA121	−3.49	SMP2	Rs_134737	RsLEA65	3.22
LEA2.5	Rs_140027	RsLEA212	−3.75	SMP2	Rs_134736	RsLEA64	2.98
LEA2.5	Rs_149607	RsLEA103	−4.03	SMP2	Rs_156298	RsLEA83	2.45
LEA3.1	Rs_153025	RsLEA80	2.23	SMP3	Rs_140941	RsLEA71	9.22
LEA3.1	Rs_125374	RsLEA53	−2.00	SMP3	Rs_106559	RsLEA34	5.99

## Data Availability

The completed *R. serbica* de novo transcriptome database is available at: https://zenodo.org/record/6341873#.YijgJ_7MJPY, accessed on 22 March 2022 (10.5281/zenodo.6341873) and translated into amino acid sequences at: https://zenodo.org/record/6340979#.YiitWP7MJPY, accessed on 22 March 2022 (10.5281/zenodo.6340979). The sequence data from this article can be found in the Short Read Archive database at NCBI under accession numbers SRR18015613 and SRR18015612 (bioproject accession no. PRJNA806723 and sample accession no. SAMN25859880).

## Supplementary Materials

The following are available online at https://www.mdpi.com/article/10.3390/ijms23073547/s1:
