# Supplementary material for: In Silico Characterisation of the Late Embryogenesis Abundant (LEA) Protein Families and Their Role in Desiccation Tolerance in Ramonda serbica Panc
Source: Int J Mol Sci. 2022 Mar 24;23(7):3547. doi: 10.3390/ijms23073547 (PMC8998581; doi:10.3390/ijms23073547)
Supplement: Supplementary file 1 [file ijms-23-03547-s001.zip › Supplementary Figure S10.pdf]

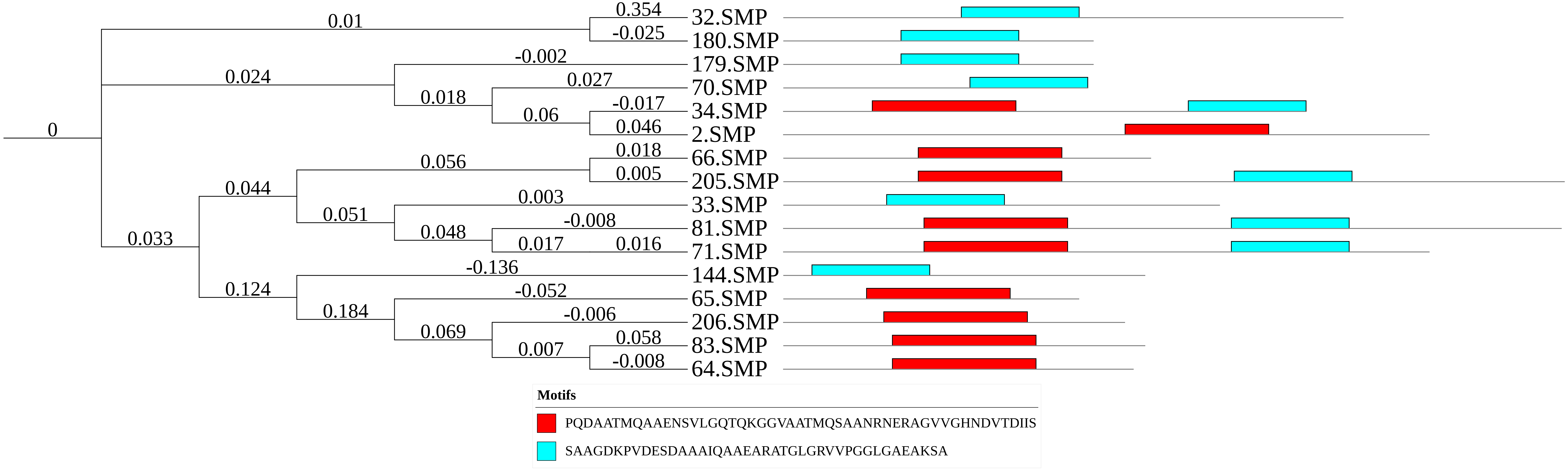
0
0.01
0.354
-0.025
32.SMP
180.SMP
0.024
-0.002
179.SMP
0.018
0.027
70.SMP
0.06
-0.017
34.SMP
0.046
2.SMP
0.033
0.044
0.056
0.018
66.SMP
0.005
205.SMP
0.051
0.003
33.SMP
0.048
-0.008
81.SMP
0.017
0.016
71.SMP
0.124
-0.136
144.SMP
0.184
-0.052
65.SMP
0.069
-0.006
206.SMP
0.007
0.058
83.SMP
-0.008
64.SMP
Motifs
PQDAATMQAAENSVLGQTQKGGVAATMQSAANRNERAGVVGHNDVTDIIS
SAAGDKPVDESDAAAIQAAEARATGLGRVVPGGLGAEAKSA
