# Supplementary material for: In Silico Characterisation of the Late Embryogenesis Abundant (LEA) Protein Families and Their Role in Desiccation Tolerance in Ramonda serbica Panc
Source: Int J Mol Sci. 2022 Mar 24;23(7):3547. doi: 10.3390/ijms23073547 (PMC8998581; doi:10.3390/ijms23073547)
Supplement: Supplementary file 1 [file ijms-23-03547-s001.zip › Supplementary Figure S5.pdf]

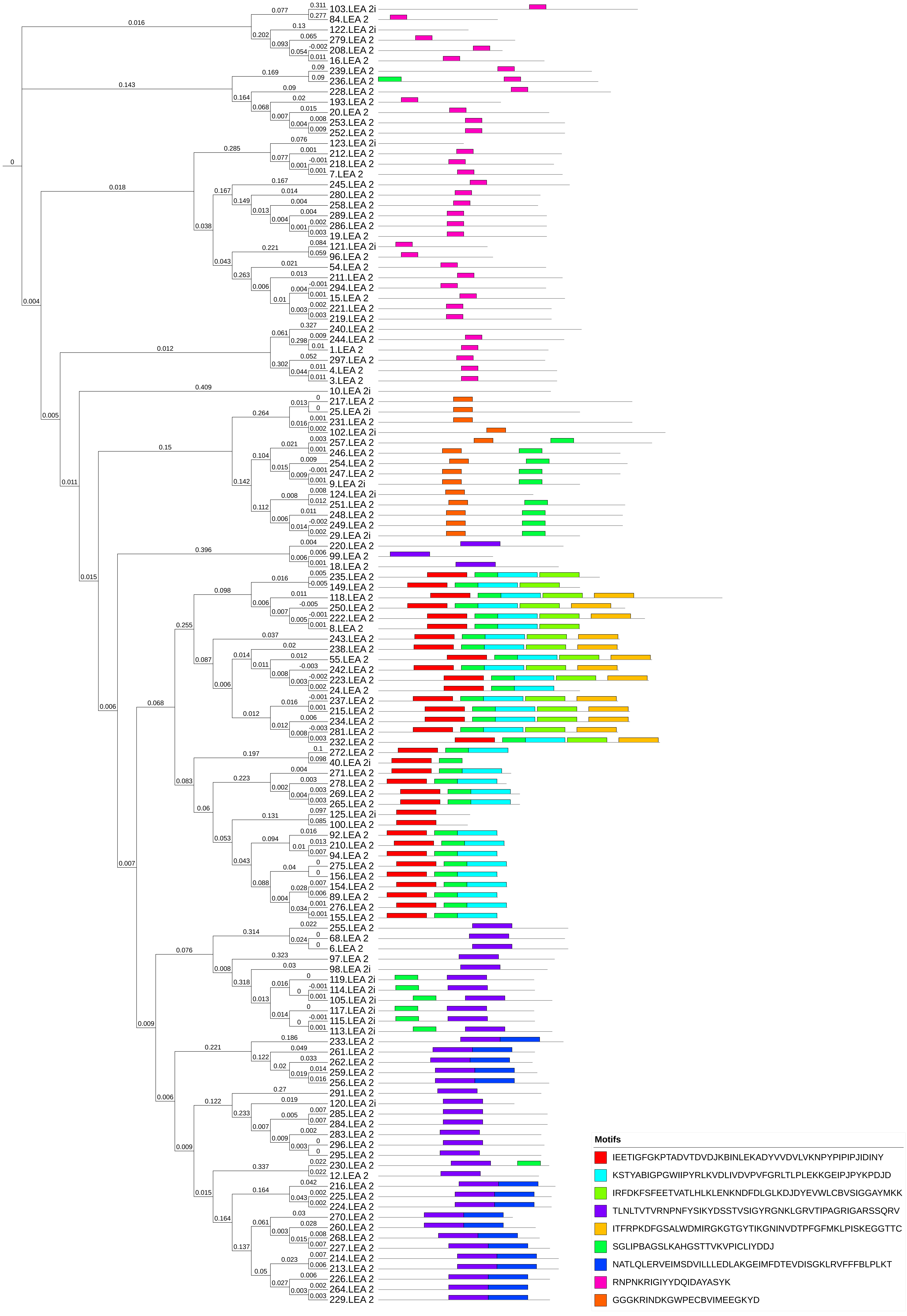
103.LEA 2i
84.LEA 2
122.LEA 2i
279.LEA 2
208.LEA 2
16.LEA 2
239.LEA 2
236.LEA 2
228.LEA 2
193.LEA 2
20.LEA 2
253.LEA 2
252.LEA 2
123.LEA 2i
212.LEA 2
218.LEA 2
7.LEA 2
245.LEA 2
280.LEA 2
258.LEA 2
289.LEA 2
286.LEA 2
19.LEA 2
121.LEA 2i
96.LEA 2
54.LEA 2
211.LEA 2
294.LEA 2
15.LEA 2
221.LEA 2
219.LEA 2
240.LEA 2
244.LEA 2
1.LEA 2
297.LEA 2
4.LEA 2
3.LEA 2
10.LEA 2i
217.LEA 2
25.LEA 2i
231.LEA 2
102.LEA 2i
257.LEA 2
246.LEA 2
254.LEA 2
247.LEA 2
9.LEA 2i
124.LEA 2i
251.LEA 2
248.LEA 2
249.LEA 2
29.LEA 2i
220.LEA 2
99.LEA 2
18.LEA 2
235.LEA 2
149.LEA 2
118.LEA 2
250.LEA 2
222.LEA 2
8.LEA 2
243.LEA 2
238.LEA 2
55.LEA 2
242.LEA 2
223.LEA 2
24.LEA 2
237.LEA 2
215.LEA 2
234.LEA 2
281.LEA 2
232.LEA 2
272.LEA 2
40.LEA 2i
271.LEA 2
278.LEA 2
269.LEA 2
265.LEA 2
125.LEA 2i
100.LEA 2
92.LEA 2
210.LEA 2
94.LEA 2
275.LEA 2
156.LEA 2
154.LEA 2
89.LEA 2
276.LEA 2
155.LEA 2
255.LEA 2
68.LEA 2
6.LEA 2
97.LEA 2
98.LEA 2i
119.LEA 2i
114.LEA 2i
105.LEA 2i
117.LEA 2i
115.LEA 2i
113.LEA 2i
233.LEA 2
261.LEA 2
262.LEA 2
259.LEA 2
256.LEA 2
291.LEA 2
120.LEA 2i
285.LEA 2
284.LEA 2
283.LEA 2
296.LEA 2
295.LEA 2
230.LEA 2
12.LEA 2
216.LEA 2
225.LEA 2
224.LEA 2
270.LEA 2
260.LEA 2
268.LEA 2
227.LEA 2
214.LEA 2
213.LEA 2
226.LEA 2
264.LEA 2
229.LEA 2
Motifs
IEETIGFGKPTADVTDVDJKBINLEKADYVVDVLVKNPYPIPIPJIDINY
KSTYABIGPGWIIPYRLKVDLIVDVPVFGRLTLPLEKKGEIPJPYKPDJD
IRFDKFSFEETVATLHLKLENKNDFDLGLKDJDYEVWLCBVSIGGAYMKK
TLNLTVTVRNPNFYSIKYDSSTVSIGYRGNKLGRVTIPAGRIGARSSQRV
ITFRPKDFGSALWDMIRGKGTGYTIKGNINVDTPFGFMKLPISKEGGTTC
SGLIPBAGSLKAHGSTTVKVPICLIYDDJ
NATLQLERVEIMSDVILLLEDLAKGEIMFDTEVDISGKLRVFFFBLPLKT
RNPNKRIGIYYDQIDAYASYK
GGGKRINDKGWPECBVIMEEGKYD
