# Supplementary material for: In Silico Characterisation of the Late Embryogenesis Abundant (LEA) Protein Families and Their Role in Desiccation Tolerance in Ramonda serbica Panc
Source: Int J Mol Sci. 2022 Mar 24;23(7):3547. doi: 10.3390/ijms23073547 (PMC8998581; doi:10.3390/ijms23073547)
Supplement: Supplementary file 1 [file ijms-23-03547-s001.zip › Supplementary Figure S8.pdf]

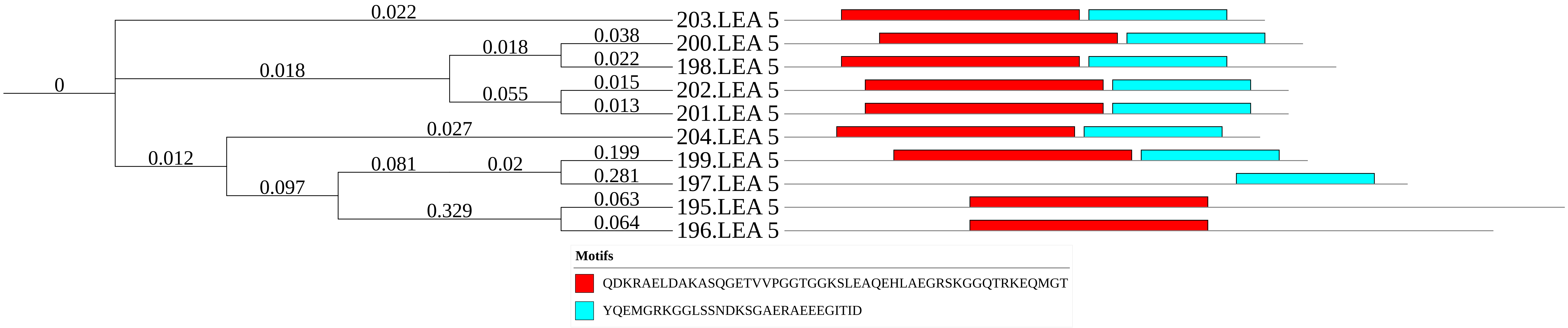
0
0.022
0.018
0.012
0.018
0.055
0.027
0.097
0.081
0.02
0.329
0.038
0.022
0.015
0.013
0.199
0.281
0.063
0.064
203.LEA 5
200.LEA 5
198.LEA 5
202.LEA 5
201.LEA 5
204.LEA 5
199.LEA 5
197.LEA 5
195.LEA 5
196.LEA 5
Motifs
QDKRAELDAKASQGETVVPGGTGGKSLEAQEHLAEGRSKGGQTRKEQMGT
YQEMGRKGGLSSNDKSGAERAEEEGITID
