# Supplementary material for: In Silico Characterisation of the Late Embryogenesis Abundant (LEA) Protein Families and Their Role in Desiccation Tolerance in Ramonda serbica Panc
Source: Int J Mol Sci. 2022 Mar 24;23(7):3547. doi: 10.3390/ijms23073547 (PMC8998581; doi:10.3390/ijms23073547)
Supplement: Supplementary file 1 [file ijms-23-03547-s001.zip › Supplementary Table S1.pdf]

Supplementary Table S1. RNAseq data quality control and transcript length distribution.

Overview of data production quality

sample	Raw reads	Clean reads	Raw data(G)	Clean data(G)	Error rate (%)	Q20 (%)	Q30 (%)	GC content (%)
HL	40137483	39608813	12	11.9	0.04	98.01	94	45.52
DL	38039070	37482969	11.4	11.2	0.04	98.02	94.1	46.01

Overview of the length distribution of transcripts and unigenes

Type	Min Length	Mean Length	Median Length	Max Length	N50	N90	total nucleotides
Transcript	201	950	683	16459	1327	452	180052827
Unigene	201	952	685	16459	1328	453	179936733

Overview of the number of transcripts and unigenes in different length intervals

Transcript length interval	200-500 bp	500-1 kbp	1-2 kbp	> 2 kbp	Total
# of Transcripts	64728	61598	44999	18131	189456
# of Unigenes	64282	61591	44999	18131	189003

The Ratio of Successfully Annotated Genes Number of Unigenes Percentage (%)

	Number of unigenes	Percentage (%)
Annotated in NR	122122	64.61
Annotated in NT	79949	42.3
Annotated in KO	43971	23.26
Annotated in Swissport	90753	48.02
Annotated in PFAM	43438	22.98
Annotated in GO	25944	13.73
Annotated in KOG	42526	22.5
Annotated in all databases	8566	4.53
Annotated in at least one database	127176	67.29
Total Unigenes	189003	100
