# Supplementary material for: In Silico Characterisation of the Late Embryogenesis Abundant (LEA) Protein Families and Their Role in Desiccation Tolerance in Ramonda serbica Panc
Source: Int J Mol Sci. 2022 Mar 24;23(7):3547. doi: 10.3390/ijms23073547 (PMC8998581; doi:10.3390/ijms23073547)
Supplement: Supplementary file 1 [file ijms-23-03547-s001.zip › Supplementary Table S5.pdf]

Supplementary Table S5. Transmembrane helical domains in *R. serbica* LEAPs.

#	Transmembrane helix domain	GRAVY index
1	V F D V H C L V L F S C S C	1.786
2	V H C L V L F S C S C C Y M	1.757
3	L G C I F A F V L G L C L I L A I T I F I V W	2.804
4	S L I S C A T V L I F L A A A L Y L L W P F D	1.813
5	Y G V I S C S F I S I F L V A A L V L L W P S	1.983
6	L I S C A T V L I F L A A A L Y L L W P F D P	1.778
7	F S W G S A L V G A A A A A A T A M M I	1.475
8	W S L F L V E A A V V L A T A L L M I V	2.315
9	G N S W S L F L V E A A V V L A A A L L M I V	1.917
10	I I C C A T V L I F L A A S L Y L L W P	2.230
11	F H Y S F N S Y I Y I Y I S L V L S I Y K F Y	0.722
12	L L Y I I L F I I F Q S A V I A A F T L T I L	2.478
13	L F L L F S F L A L L V L A V I L V I V L A V	3.357
14	C V V A T V F L L A I A A A A V A V Y F F V F	2.678
15	C L C Y V A A F V V L Q T I V I V V F S L V F	2.630
16	L Q L V Y S G L P V G I V F I P A G R I G G G	1.148
17	Y V L A F V V G F F V L F S F F A L I L L A A	2.687
18	L I C I S I A T V I V L A G L A A L T V W L L	2.591
19	I F Q I I I T I L I V V A I V V F I C W L I F	3.152
20	G L M A V G G F I L L F T V F C L I L W G A G	2.139
21	L R C I A F T A L A L I I L T A L V V L I I W	2.583
22	L Y L Y F S T I F L I L L S L I L L V Y L I L	2.591
23	L C W T I T T L I L L L I I V A A V L A I L Y	2.657
24	C C A C L L I S I F L I L F T I L L V W A I L	2.939
25	I F A F F I A L L V I S G T I T L I V W L V L	2.722
26	F W A I L F L I G I L L L A A I A A A A F Y V	2.591
27	I C L C V I A V I L G L G L L F L I L G L T V	2.987
28	I C C C Y C C L L L L I I I L A A L A F C F Y	2.791
29	L F Y Y L I T N L L T I L S L I L L V W L I L	2.339
30	I A F T A L A L I I L T V L V V L I I W A T V	2.839
31	I V W F G A I L C L L F S L L L I F F G I A T	2.570
32	C I L A A I F L T L F I A A A F I V Y F I V F	2.861
33	L Q F L Y A G N Q V G L L F I P A G M I S G G	1.013
34	V T C V F L L M L L A V I C V A G N	2.511
35	I Y S I Y A T Y V G I A C C S C T G C T I T C	1.270
36	C C A C L L I T I F L I L F T I L L I W A I L	2.957
37	I L S F H L F Q V S I I I I I I S R S I	1.950
38	A S F N R I L I V S S V S S V Y Y Y Y Y I I S	0.857
