# Supplementary figures and images for: In Silico Characterisation of the Late Embryogenesis Abundant (LEA) Protein Families and Their Role in Desiccation Tolerance in Ramonda serbica Panc
Source: Int J Mol Sci. 2022 Mar 24;23(7):3547. doi: 10.3390/ijms23073547 (PMC8998581; doi:10.3390/ijms23073547)

## Species Classification

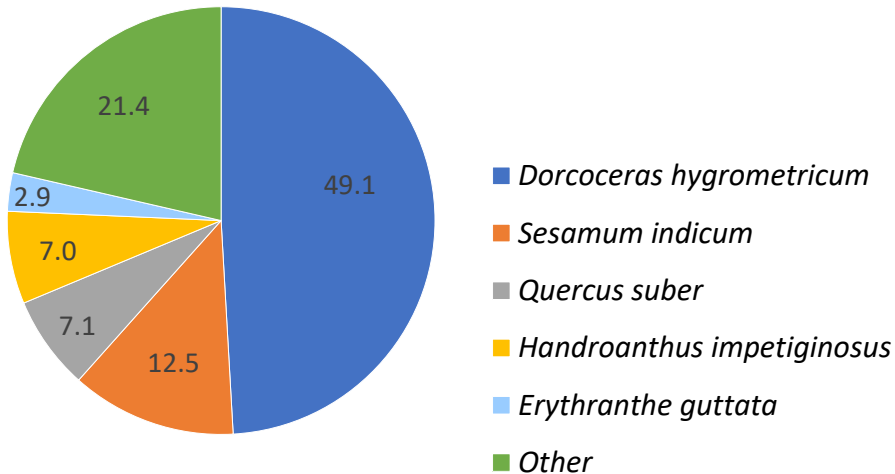

Supplement: Supplementary file 1 [file ijms-23-03547-s001.zip › Supplementary Figure S1.pdf]

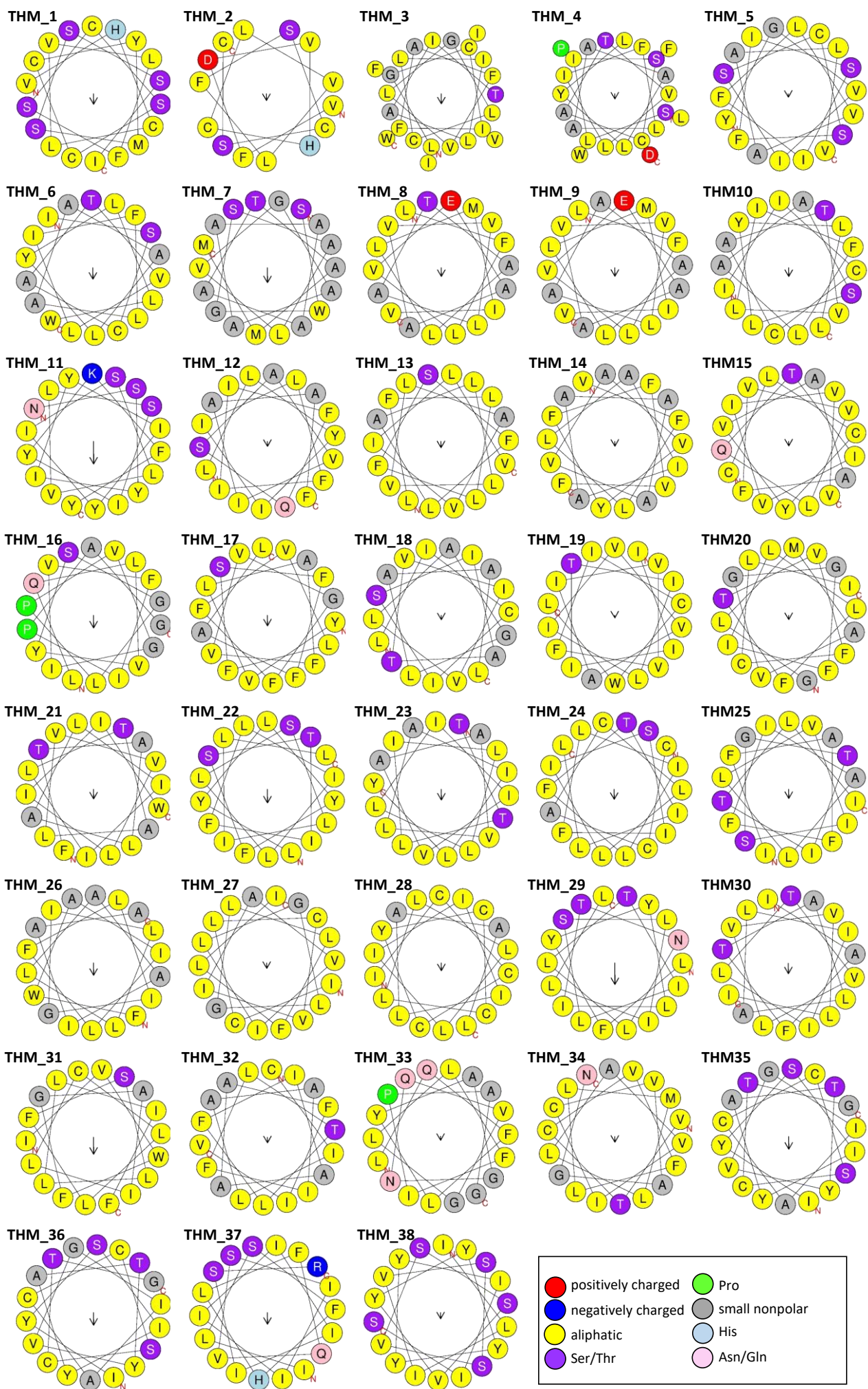

Supplement: Supplementary file 1 [file ijms-23-03547-s001.zip › Supplementary Figure S11.pdf]

**RsLEA\_86**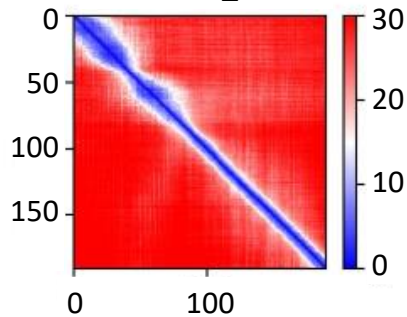**RsLEA\_211**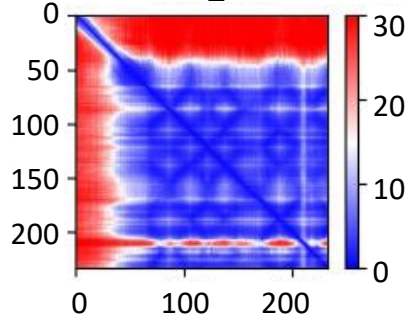**RsLEA\_80**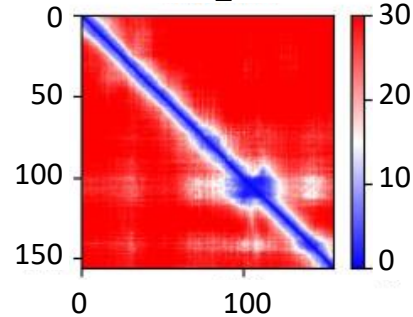**RsLEA\_301**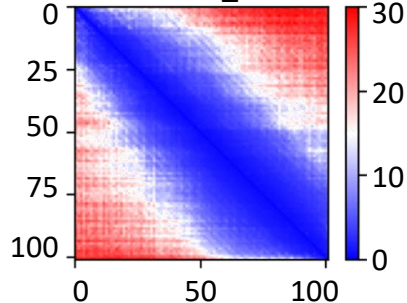**RsLEA\_188**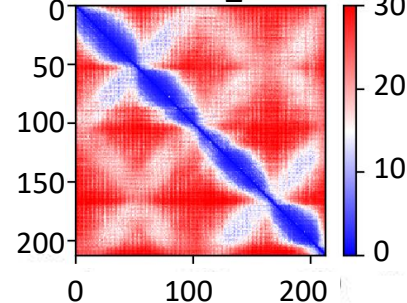**RsLEA\_202**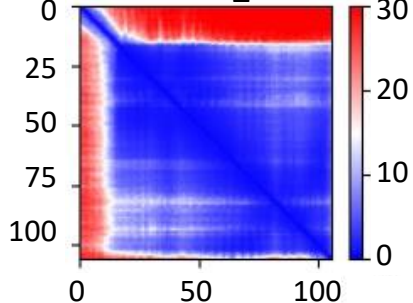**RsLEA\_275**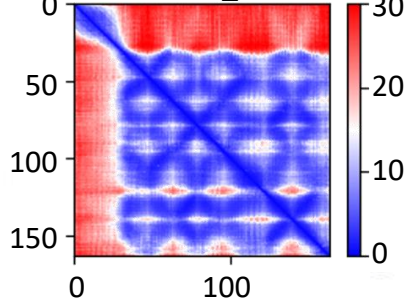**RsLEA\_139**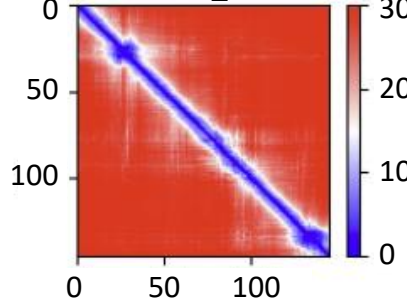**RsLEA\_55**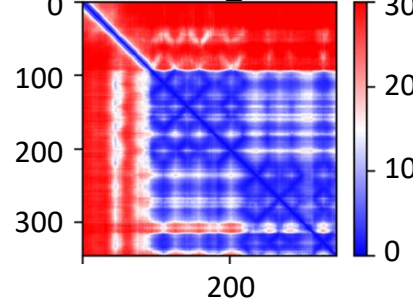**RsLEA\_66**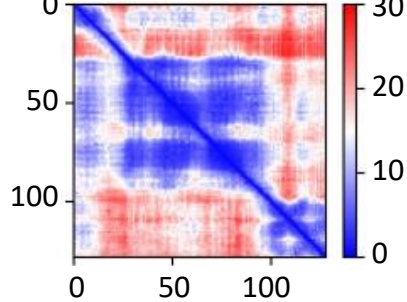**RsLEA\_71**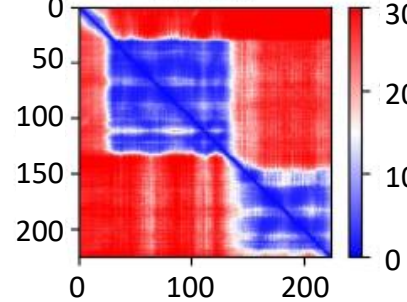

Supplement: Supplementary file 1 [file ijms-23-03547-s001.zip › Supplementary Figure S12.pdf]

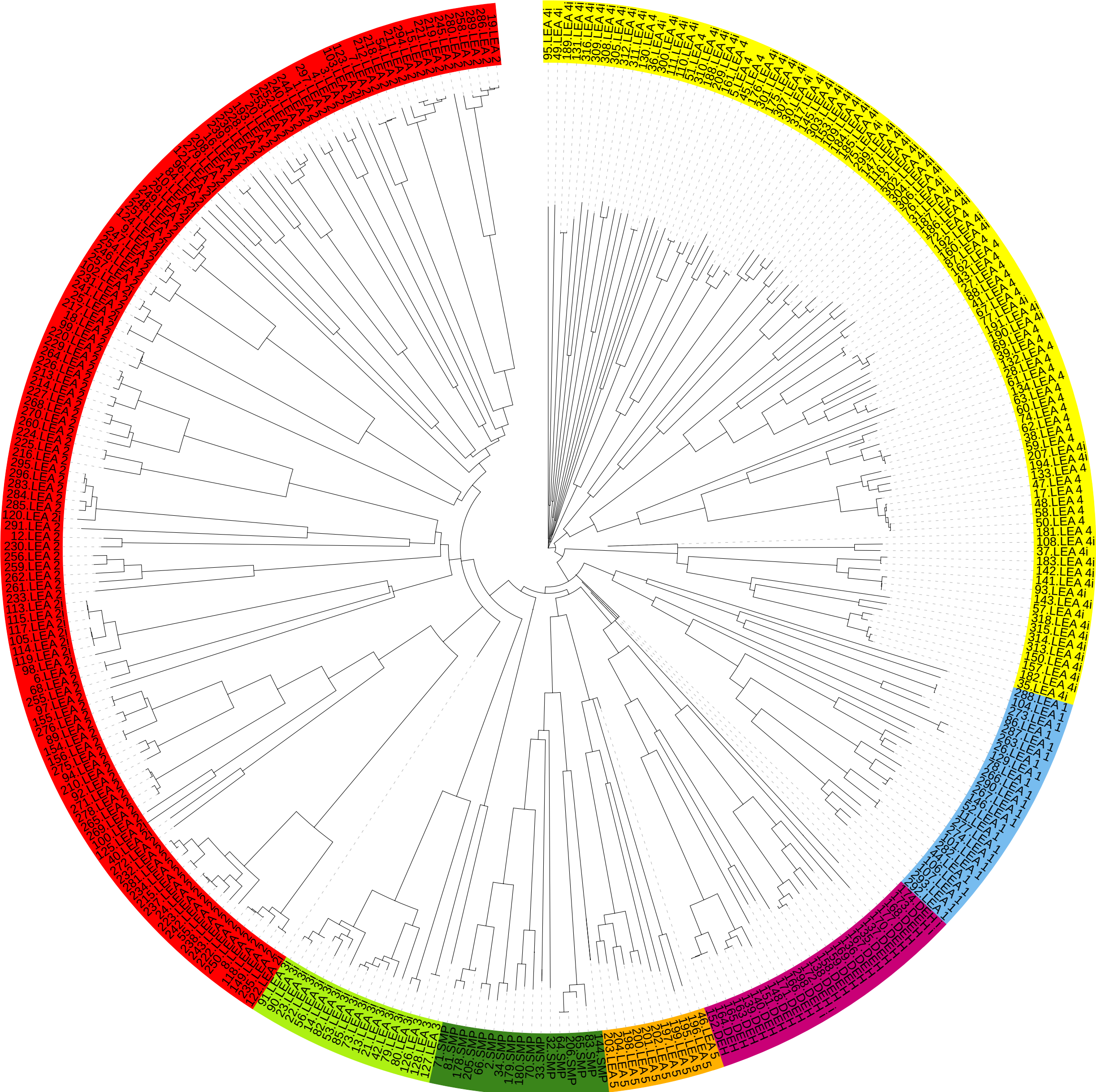

Tree scale: 0.1

Supplement: Supplementary file 1 [file ijms-23-03547-s001.zip › Supplementary Figure S2.pdf]

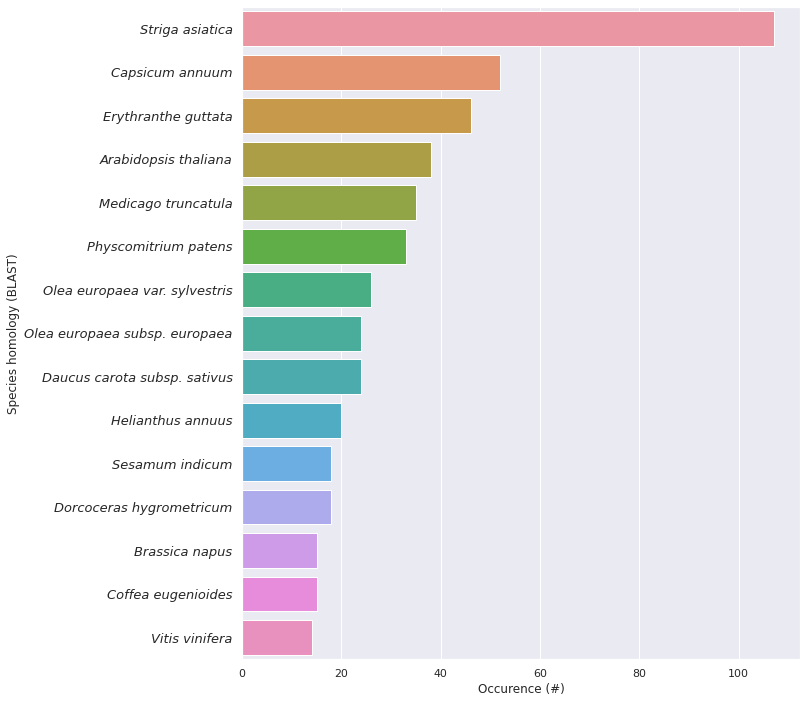

Supplement: Supplementary file 1 [file ijms-23-03547-s001.zip › Supplementary Figure S3.png]

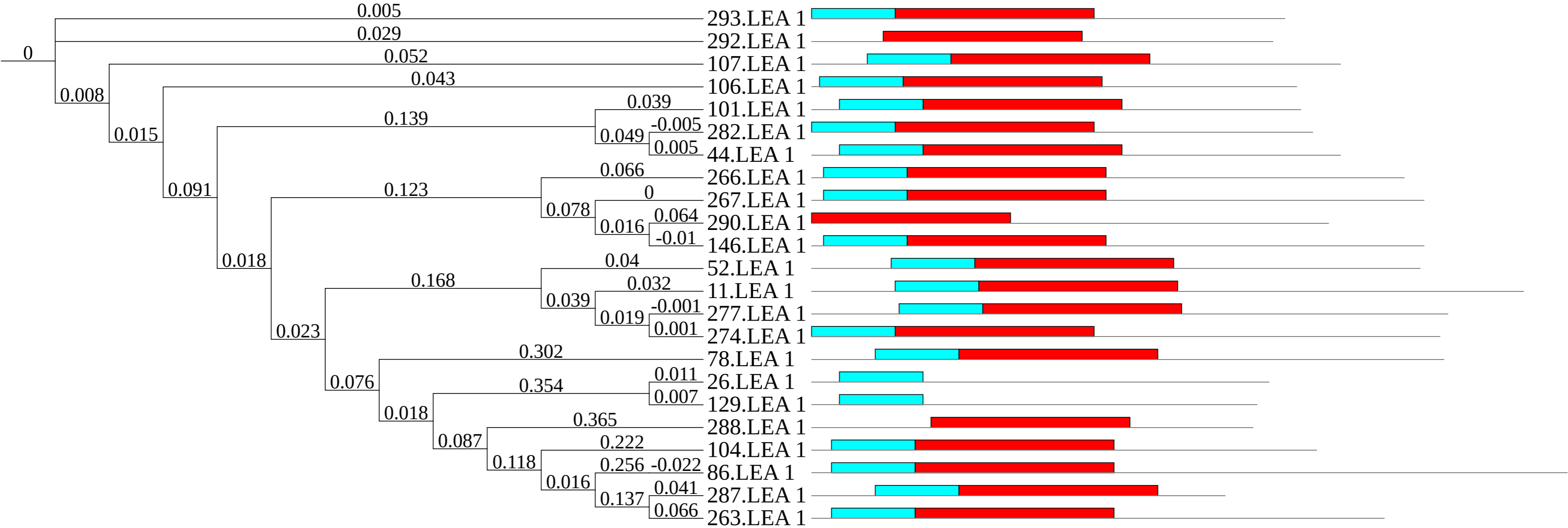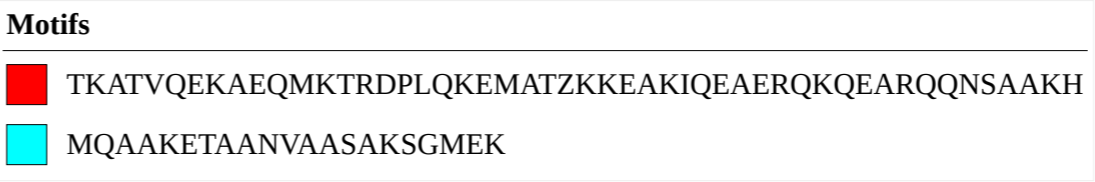

Supplement: Supplementary file 1 [file ijms-23-03547-s001.zip › Supplementary Figure S4.pdf]

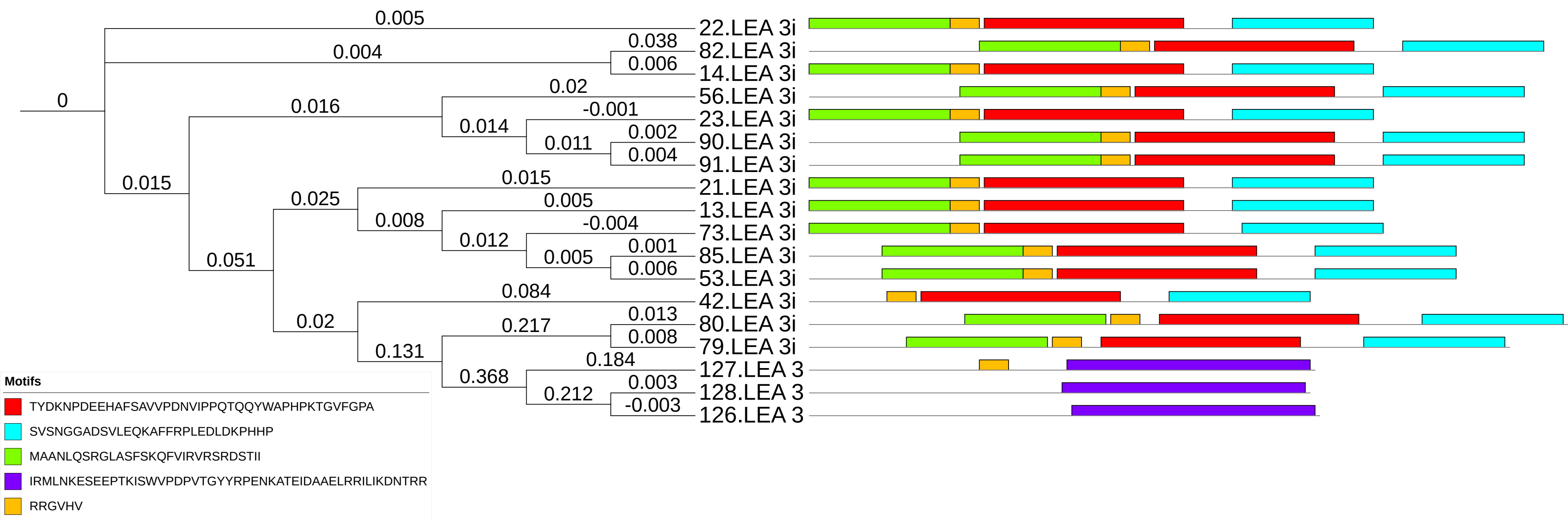

Supplement: Supplementary file 1 [file ijms-23-03547-s001.zip › Supplementary Figure S6.pdf]

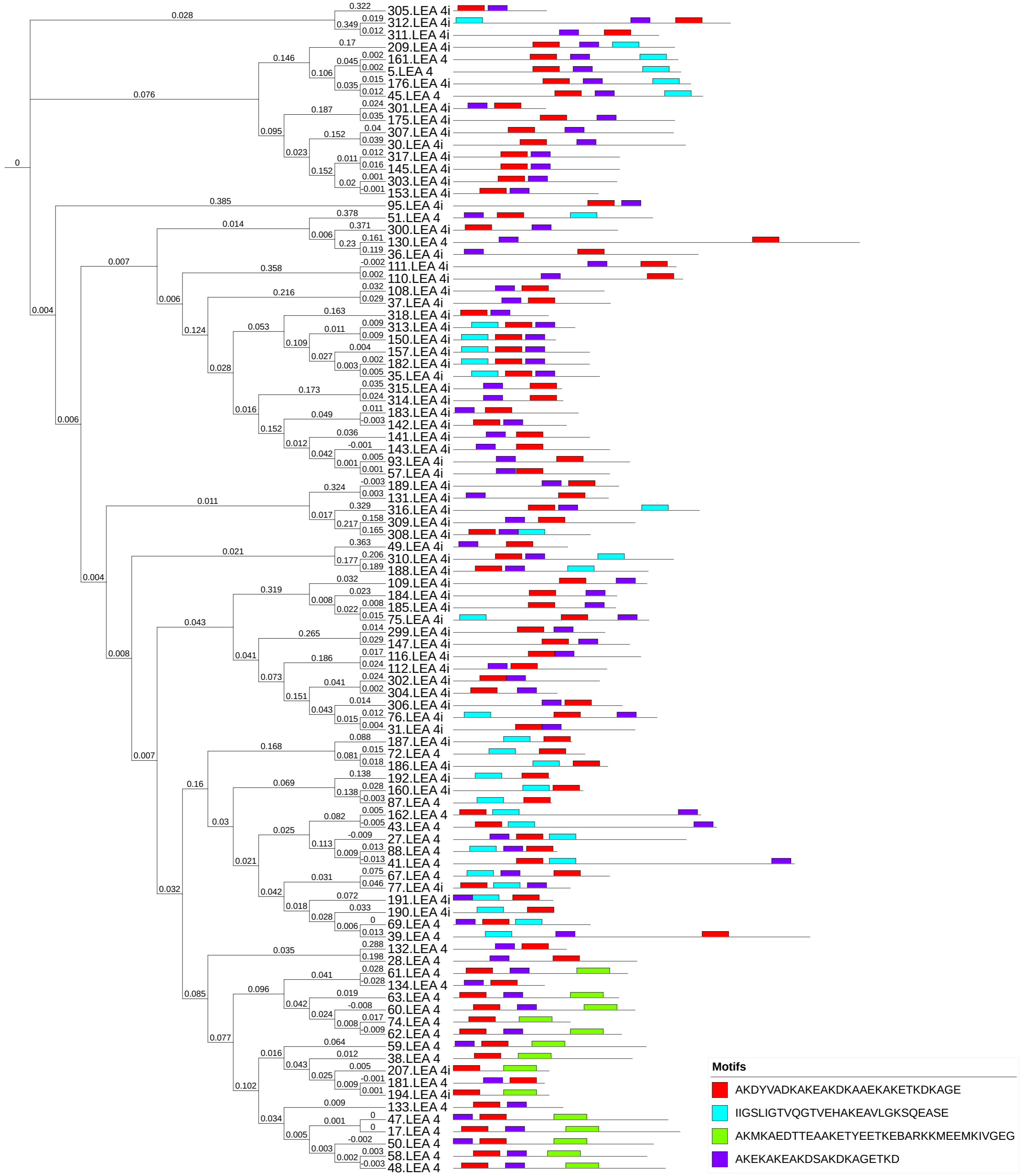

Supplement: Supplementary file 1 [file ijms-23-03547-s001.zip › Supplementary Figure S7.pdf]

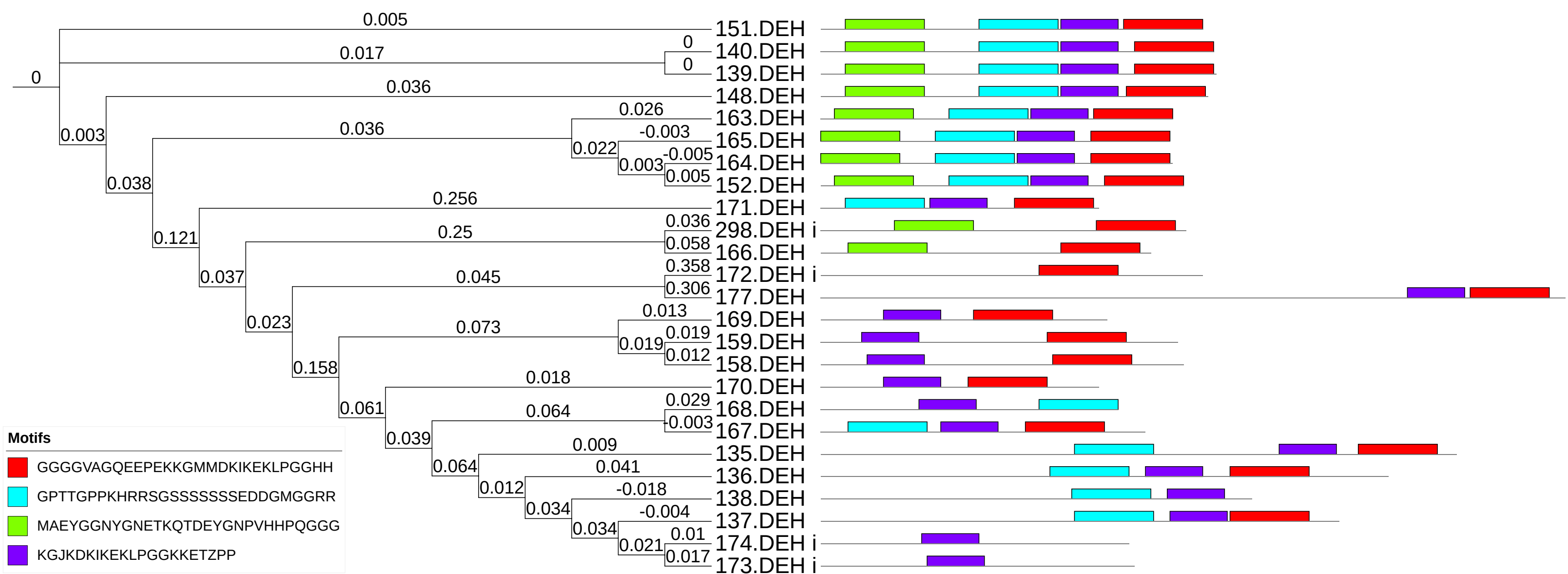

Supplement: Supplementary file 1 [file ijms-23-03547-s001.zip › Supplementary Figure S9.pdf]
